# Induction of CD4^+^CD25^+^FOXP3^+^ regulatory T cells by mesenchymal stem cells is associated with modulation of ubiquitination factors and TSDR demethylation

**DOI:** 10.1186/s13287-018-0991-1

**Published:** 2018-10-25

**Authors:** Maryam Khosravi, Ali Bidmeshkipour, José L. Cohen, Ali Moravej, Suzzan Hojjat-Assari, Sina Naserian, Mohammad Hossein Karimi

**Affiliations:** 1 Transplant Research Center, Shiraz University of Medical Sciences, Shiraz, Iran; 2 Department of Biology, Faculty of Science, Razi University, Kermanshah, Iran; 3 Université Paris-Est, UMR_S955, UPEC, F-94000 Créteil, France; 4 Inserm, U955, Equipe 21, F-94000 Créteil, France; 5 UPEC, APHP, Inserm, CIC Biothérapie, Hôpital Henri Mondor, 94010 Créteil, France; 6 Noncommunicable Diseases Research Centre, Fasa University of Medical Sciences, Fasa, Iran; 7 Institut Français de Recherche et d’Enseignement Supérieur à l’International (IFRES-INT), Paris, France; 8 Inserm, U1197, Hôpital Paul Brousse, 94807 Villejuif, France; 9 SivanCell, Alborz University of Medical Sciences, Alborz, Iran

**Keywords:** Mesenchymal stem cells, Regulatory T cells, Ubiquitination molecules

## Abstract

**Background:**

Mesenchymal stem cells (MSCs) are known for their ability to induce the conversion of conventional T cells (Tconvs) into induced regulatory T cells (iTregs) in specific inflammatory contexts. Stable Foxp3 expression plays a major role in the phenotypic and functional stability of iTregs. However, how MSCs induce stable Foxp3 expression remains unknown.

**Methods:**

We first investigated the role of cell–cell contact and cytokine secretion by bone marrow-derived MSCs (BM-MSCs) on the induction, stability, and suppressive functions of Tregs under various experimental conditions that lead to Foxp3 generation by flow cytometry and ELISA respectively. Second, we studied the effect of MSCs on TRAF6, GRAIL, USP7, STUB1, and UBC13 mRNA expression in CD4^+^ T cells in correlation with the suppressive function of iTregs by real-time PCR; also, we investigated Foxp3 Treg-specific demethylated region (TSDR) methylation in correlation with Foxp3 stability by the high-resolution melting technique. Third, we studied the effect of ex-vivo-expanded BM-MSCs on the induction of transplant tolerance in a model of fully allogeneic skin transplantation. We further analyzed the cytokine secretion patterns in grafted mice as well as the mRNA expression of ubiquitination genes in CD4^+^ T cells collected from the spleens of protected mice.

**Results:**

We found that in-vitro MSC-induced Tregs express high mRNA levels of ubiquitination genes such as TRAF6, GRAIL, and USP7 and low levels of STUB1. Moreover, they have enhanced TSDR demethylation. Infusion of MSCs in a murine model of allogeneic skin transplantation prolonged allograft survival. When CD4^+^ T cells were harvested from the spleens of grafted mice, we observed that mRNA expression of the Foxp3 gene was elevated. Furthermore, Foxp3 mRNA expression was associated with increased TRAF6, GRAIL, UBC13, and USP7 and decreased STUB1 mRNA levels compared with the levels observed in vitro.

**Conclusions:**

Our data suggest a possible ubiquitination mechanism by which MSCs convert Tconvs to suppressive and stable iTregs.

**Electronic supplementary material:**

The online version of this article (10.1186/s13287-018-0991-1) contains supplementary material, which is available to authorized users.

## Background

Bone marrow-derived mesenchymal stem cells (BM-MSCs) are capable of modulating the immune response in vitro and in vivo during anti-tumor immune responses, autoimmunity, graft rejection, and graft-versus-host disease (GVHD) [14, 24, 29, 30]. The immunomodulatory properties of MSCs depend on the production of inhibitory molecules and cytokines (i.e., IL-10, TGF-β, HLA-G, IDO, and PGE2), and on the induction of Tregs and/or tolerogenic dendritic cells (tDCs) [24, 46] that are cell–cell contact independent. It has been shown that in the presence of MSCs, dendritic cells (DCs), naïve and effector T cells (TH1, TH2, and TH17), and natural killer cells (NKs) could convert into a regulatory phenotype [49]. In particular, the maturation, function, and differentiation of DCs are affected by MSCs through the reduction in the surface membrane expression of MHC class II costimulatory molecules and reduced secretion of IL-12 and tumor necrosis factor alpha (TNF-α) [19]. MSC-induced tDCs trigger CD4^+^ T cells to assume an anti-inflammatory phenotype [17, 33]. The tolerogenic factors produced by tDCs have been shown to include IL-10, TGF-β, retinoic acid (RA), and membrane receptors such as immunoglobulin-like transcript 3 (ILT3) and programmed death-1 ligand 1 (PDL-1) [23, 28, 51]. Furthermore, it has been demonstrated that ILT receptors, which are found on tDCs, can stimulate Treg differentiation [25, 26, 59]. Recently, we and others reported that MSC-induced DCs express ILT3, ILT4, PDL-1, and IDO, molecules that enhance the differentiation of suppressive Tregs [18, 38–40]. These tDCs can induce or enhance the suppressive function of existing Tregs and convert activated T cells into induced Tregs [36].

Tregs are crucial factors in maintaining self-tolerance and homeostasis [53]. These cells can be categorized as either natural Tregs (nTregs) that develop in the thymus during embryonic states or induced Tregs (iTregs) that develop from effector T cells in the periphery in particular inflammatory settings [34]. Previous works have shown that MSCs favor iTreg conversion and enhance iTreg and nTreg expansion and suppressive functions both in vitro and in vivo [7]. Stable Foxp3 expression plays a major role in the phenotypic and functional stability of iTregs. Indeed, under certain inflammatory conditions, iTregs lose their Foxp3 expression and are converted into effector T cells [52]. It has already been shown that hypomethylation of the Treg-specific demethylated region (TSDR) in the Foxp3 promoter is required for stable Foxp3 expression [31]. The TSDR is completely demethylated in nTregs, partially methylated in iTregs, and completely methylated in effector T cells [31]. Hence, removal of methyl groups from the TSDR enhances the binding of transcription factors to the TSDR and increases Foxp3 expression. The role of TSDR methylation in Foxp3 stability is supported by TSDR-null Tregs, which have lost their Foxp3 expression [31].

Tregs hence affect the suppressive function of these cells. This process occurs through several mechanisms, including the action of ubiquitination enzymes such as TRAF6 (tumor necrosis factor receptor-associated factor 6), GRAIL (gene related to anergy in lymphocytes), USP7 (ubiquitin-specific protease 7), UBC13 (ubiquitin-conjugating enzyme 13), and STUB1 (STIP1 homology and U-box-containing protein 1), all of which have been demonstrated to play key roles in Treg suppressive function [11].

Ubiquitin-activating enzyme (E1) uses ATP to bind to Ub (ubiquitin). The activated Ub is transferred to a ubiquitin-conjugating enzyme (E2). The E2 is brought to the substrate by binding to a ubiquitin-protein ligase (E3) that binds to both the E2 and the substrate. Once bound to an E3, the E2 either directly transfers Ub to the substrate or transfers it through a thiol ester linkage to the E3, which then transfers it to the substrate. Attachment of Ub to lysine 48 (K48) generally results in proteasome degradation, whereas attachment of Ub to K63 results in protein activation, localization, and signaling [62].

TRAF6 is a K63 E3 ubiquitin ligase that plays an important role in the maintenance of Foxp3 expression and suppressive functions in Tregs both by inhibiting the conversion of naïve T cells to Th17 cells and by promoting the conversion of T cells to iTregs [42]. As K63-type ubiquitination can dictate the intracellular distribution and signaling activity of numerous target proteins, it is anticipated that modification of Foxp3 by TRAF6 promotes the nuclear localization and gene-regulating activity of key transcription factors. Indeed, immune staining of cell lines and primary T cells revealed that under conditions of TRAF6 deficiency, Foxp3 shows a diffuse distribution throughout the cell, whereas TRAF6-competent cells, as expected, display primarily nuclear Foxp3 staining [2]. UBC13 is an E2 ubiquitin ligase that acts by increasing the activity of TRAF6 [60]. GRAIL is a K48 E3 ubiquitin ligase that is highly expressed in Tregs but not in conventional T cells. Forced expression of GRAIL by conventional T cells was shown to favor their conversion to iTregs [35]. Indeed, GRAIL maintains the Treg-suppressive phenotype by degradation of NFATC1 via proteasomes, thus inhibiting IL-17 and IL-21 gene expression in Tregs [45]. USP7 is a deubiquitination enzyme that tends to detach ubiquitin from Foxp3 and thereby maintains a high level of Foxp3 in the cells [58]; thus, USP7 plays an important role in the suppressive function of Tregs [58]. STUB1 is one of the K48 E3 ubiquitin ligase enzymes that promotes Foxp3 degradation by proteasomes, thus reducing the amount of Foxp3 in Tregs. Previous investigations showed that STUB1 expression is decreased in functional and suppressive Tregs [12].

Although the regulatory effect of MSCs on the immune system has been well studied, the molecular mechanism underlying this phenomenon is still an unresolved question, and, to our knowledge, the effects of MSCs on ubiquitination gene expression in Tregs have not yet been explored. In this work, we first investigated the role of cell–cell contact and cytokine secretion by BM-MSCs on the induction, stability, and suppressive functions of Tregs under various experimental conditions that lead to Foxp3 generation, including the following conditions: coculture of MSCs with allogeneic CD4^+^CD25^−^ T cells (MSC + TC); coculture of MSCs with autologous DCs and allogeneic CD4^+^CD25^−^ T cells in the presence of lipopolysaccharide (LPS) (MSC + MLR + LPS); coculture of MSCs with autologous DCs and allogeneic CD4^+^CD25^−^ T cells in the absence of LPS (MSC + MLR); and, finally, coculture of allogeneic CD4^+^CD25^−^ T cells and MSC-treated DCs (MSC-DC).

Second, we studied the effect of MSCs on TRAF6, GRAIL, USP7, STUB1, and UBC13 mRNA expression in CD4^+^ T cells in correlation with the suppressive function of iTregs, and investigated Foxp3 TSDR methylation in correlation with Foxp3 stability. Third, we studied the effect of ex-vivo-expanded BM-MSCs on the induction of transplant tolerance in a model of fully allogeneic skin transplantation. We further analyzed the cytokine secretion patterns in grafted mice as well as the mRNA expression of ubiquitination genes in CD4^+^ T cells collected from the spleens of protected mice.

We demonstrate for the first time that MSCs can enhance the suppressive phenotype and the stability of Tregs by regulating the mRNA expression of ubiquitination genes and TSDR demethylation both in vitro and in vivo.

## Methods

### MSC culture

MSCs were collected by flushing the femoral and tibial bone marrow of 6–8-week-old female BALB/c mice. The mice were purchased from the central animal laboratory of Shiraz University of Medical Sciences, Iran. This research was approved by the Committee on Ethics in Animal Experiments (CEEA) of Shiraz Medical Sciences University. All methods and procedures were performed in accordance with the relevant guidelines and regulations. The cells were cultured in 25-cm^2^ flasks in Dulbecco’s Modified Eagle’s Medium (DMEM) containing low glucose, GlutaMAX I, 10% heat-inactivated FBS, 1% penicillin, and streptomycin (all from Gibco, Germany). The cells were incubated at 37 °C in a 5% CO_2_ atmosphere. Nonadherent cells were removed every 8 h; pure MSCs were obtained after 4–5 weeks. Murine MSCs derived from bone marrow express surface markers such as CD44 and Sca-1 and do not express specific hematopoietic markers such as CD34 and CD45. The presence of these markers was tested using PE-conjugated anti-Sca-1, anti-CD44, and anti-CD45 antibodies and FITC-conjugated anti-CD34 obtained from eBioscience, USA. Unstained cells and cells of the proper isotype were used as controls, and the data were analyzed using FlowJo software. The second passage of MSCs was used for experiments.

BM-MSCS can differentiate into osteocytes or adipocytes when cultured under appropriate differentiation conditions (DMEM supplemented with 0.5 μM ascorbyl phosphate, 1 μM dexamethasone, and 200 μM indomethacin) for 10 days. Cells that had been treated in this manner were stained with 0.5% Oil Red for 10 min. To differentiate isolated cells into osteocytes, they were cultured in a specific differentiation medium (DMEM supplemented with 0.5 μM ascorbyl phosphate, 10 mM β-glycerophosphate, and 1 μM dexamethasone) for 21 days and stained with 1% Alizarin Red for 10 min.

### Isolation of DCs and CD4^+^ T cells

**Fig. 1:**
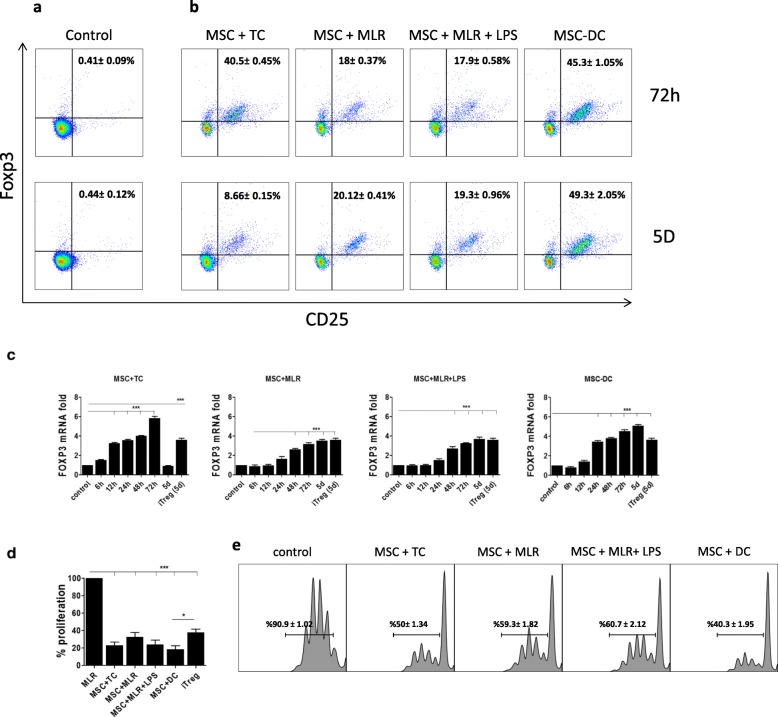
MSCs can convert conventional T cells into Foxp3-expressing Tregs with strong immunosuppressive capacity. CD4^+^ effector T cells and DCs isolated and cultured with allogeneic MSCs in Transwell system under four conditions as described in Methods. **a** CD4^+^CD25^−^ purity before culturing with MSCs. **b** CD4^+^ T cells harvested, and CD4^+^CD25^+^FOXP3^+^ regulatory T cells measured by flow cytometry after 72 h and 5 days of coculturing with MSCs. **c** Total mRNA extracted from MSC-induced Tregs after 6 h, 12 h, 24 h, 48 h, 72 h, and 5 days of coculture, and FOXP3 mRNA expression measured by real-time PCR. Allogeneic MLR performed; CD4^+^CD25^−^ effector T cells isolated 5 days after MLR used as negative control, and TGF-β-induced Treg cells used as positive control. mRNA samples normalized to expression of GAPDH and compared with negative control. **d** For suppression assay, MSC-cultured T cells isolated after 72 h and added to T cells that had been stimulated by allogeneic DCs. After 48 h, BrdU used to measure proliferation of CD4^+^ T cells; proliferation of these cells compared with that of MLR control group consisting of T cells cultured in presence of allogeneic DCs. **e** MSCs and T cells cocultured for 3 days. Total T cells (cells in suspension) collected and CD4^+^CD25^+^ population isolated via Treg isolation kit. These newly induced Tregs were further put in new MLR encountering freshly isolated CD4^+^CD25^−^ responder cells in a 1/10 ratio (1 Treg/10 Tconvs) in presence of anti-CD3 and anti-CD28 activation antibodies. Suppressive assay then investigated by analysis of CFSE expression. Data presented as mean ± SEM; *n* = 2 (**a**) and *n* = 4 (**b, c**) independent experiments. Significant results: *p < 0.05; ***p < 0.001. d day, DC dendritic cell, iTreg induced Treg, LPS lipopolysaccharide, MSC mesenchymal stem cell, MSC-DC MSC-treated DC, MLR mixed lymphocyte reaction, TC allogeneic CD4^+^CD25^−^ T cells

The spleens of BALB/c mice were removed for DC isolation. The most practical way to enrich the DC fraction is to use a density gradient. Nycodenz™ has successfully been used for enrichment of DCs obtained from various sources [41]. After the cells were fractionated using Nycodenz™, a CD11c-positive selection kit (Miltenyi Biotechnology, Germany) was used to further enrich for DCs according to the manufacturer’s guide. A CD4^+^ T-cell negative selection kit (Miltenyi Biotechnology, Germany) was used to isolate total CD4^+^ T cells from the spleens of C57BL/6 mice. CD4^+^CD25^+^ cells were removed from the CD4^+^ T-cell pool, which showed more than 95% purity of CD4^+^CD25^−^ T cells, using the CD4^+^CD25^+^ Regulatory T Cell Isolation Kit (Miltenyi Biotechnology, Germany) (Fig. 1a). The resulting CD4^+^CD25^−^ T cells were cultured in the presence of MSCs. The CD4^+^CD25^+^ Regulatory T Cell Isolation Kit was used to isolate Tregs following culture with MSCs. The magnetic-activated cell sorting (MACS) method was used in all cell isolations. The isolation of DCs and T cells from coculture in the presence of MSCs is based on the biological capacity of MSCs to adhere to plastic plates; however, DCs and T cells always stay in suspension, hence we isolated them with gentle aspiration using a pipette.

Staining with PE-conjugated anti-CD11c antibody to identify DCs and with PE-conjugated anti-CD4 antibody to identify T cells showed that the cell populations were more than 90% pure. Moreover, annexin–PI staining demonstrated the high viability of these cells.

### In-vitro study design

In this work, we reproduced several previously studied MSC culture models that have been shown to lead to Treg induction [6, 38]. In the first set of experiments, we sought to determine whether MSCs induce Tregs under all these conditions via the same mechanisms. It is possible that MSCs influence T cells directly or indirectly. In the direct mechanism, the effect of MSCs on T cells might occur through cell–cell contact or through the release of soluble factors; the effect of MSCs on T cells might also occur indirectly via modulation of antigen-presenting cells (APCs) such as DCs, resulting in altered cytokine expression and impaired antigen presentation [16].

With the foregoing possibilities in mind, we considered four experimental conditions:

1. In the MSC + TC condition, we added 2 × 10^6^ CD4^+^CD25^−^ T cells directly to 2 × 10^5^ allogeneic MSCs.

The rationale for using condition 1 was to investigate the direct effects of MSCs on Treg induction and their suppressive phenotype.

2. In the MSC + MLR condition, we added 2 × 10^6^ CD4^+^CD25^−^ T cells (C57BL/6) and 2 × 10^5^ DCs (BALB/c) to 2 × 10^5^ MSCs (BALB/c).
3. In the MSC + MLR + LPS condition, we added 2 × 10^6^ CD4^+^CD25^–^ T cells (C57BL/6) and 2 × 10^5^ DCs (BALB/c) to 2 × 10^5^ MSCs (BALB/c). LPS (200 ng/ml) (Sigma, USA) was then directly added to each well.

For conditions 2 and 3, T cells were separated from DCs using a CD4^+^ isolation kit and MACS.

The rationale for choosing conditions 2 and 3 was that it has already been shown that LPS increases Notch ligand expression on DCs [1] and that these ligands are needed for MSCs to induce functional tolerogenic DCs. Furthermore, TLR stimulation can enhance the suppressive features of MSCs; however, the latter findings are controversial [32]. Previous investigations have also shown that LPS has different effects on TH17, TH2, TH1, and Tregs depending on anti-inflammatory or proinflammatory conditions [37]. Therefore, we performed MLR in the presence of MSCs with or without LPS to assess whether or not LPS treatment enhances the immunosuppressive feature of MSCs.

4. In the MSC-DC condition, 2 × 10^6^ freshly isolated DCs (BALB/c) were first cultured with 2 × 10^5^ autologous MSCs for 24 h. After 24 h, 2 × 10^5^ of these DCs were isolated and irradiated (30 Gy) and added to 2 × 10^6^ freshly isolated CD4^+^CD25^−^ allogeneic T cells. The rationale for choosing this condition was to investigate the indirect effects of MSCs on Treg induction and their suppressive phenotype. The tolerogenic factors produced by tDCs are IL-10, TGF-β, retinoic acid, and several membrane receptors such as immunoglobulin-like transcript3 (ILT3) and programmed death-1 ligand 1 (PDL-1) [23, 28, 51]. It has been shown that ILT receptors found on tDCs can stimulate Treg differentiation [25, 26, 59]. Moreover, it was recently reported that MSC-treated DCs express ILT3, ILT4, PDL-1, and IDO and therefore could enhance the differentiation of suppressive Tregs [18, 38–40]. tDCs can induce or enhance the suppressive function of existing Tregs and convert activated T cells into Tregs [36].

MSC-induced Tregs were isolated using a CD4^+^CD25^+^ Treg isolation kit for biological investigations in all conditions

### Coculture and Transwell culturing of T cells and MSCs

The second passage of BALB/c MSCs was seeded into six-well plates, and the MSCs were incubated for 3 h in complete DMEM. Freshly isolated T cells from C57BL/6 mice were then added to the MSCs at a 1:10 ratio. All coculture experiments were performed in DMEM containing 10% FBS, 1% penicillin, and 1% streptomycin. T cells were harvested after 6 h, 12 h, 24 h, 48 h, 72 h, and 5 days of coculture. Transwell experiments were also performed using the same cell ratios and the same conditions as already described; in all Transwell conditions, CD4^+^CD25^−^ T cells and/or DCs were cultured in the lower chambers and MSCs were seeded in the upper chambers of Transwell plates (0.8-μm pore size membrane; SPL, USA).

### Mixed lymphocyte reaction and negative control

DCs were irradiated (30 Gy), washed three times in PBS, and resuspended in RPMI supplemented with 10% heat-inactivated FBS, 1% penicillin, and 1% streptomycin. The mixed lymphocyte reaction (MLR) was conducted in 96-well round-bottom cell culture plates (Nunc, Denmark). As stimulator cells, 10^5^ untreated DCs (BALB/c) were cocultured with 10^6^ CD4^+^CD25^–^ T cells (C57BL/6) as responder cells in a total volume of 200 μl. After 5 days, CD4^−^CD25^+^ effector T cells were isolated using a CD4^+^CD25^−^ T-cell isolation kit and used as a negative control.

### TGF-β-induced Tregs and positive control

To generate iTregs, we used Fantini et al.’s protocol [22]. Briefly, CD4^+^CD25^−^ T cells were isolated. A 24-well cell culture plate was coated with anti-CD3 antibody at 10 μg/ml in PBS for 2 h at 37 °C. Next, the plate was washed once with PBS before plating 1 ml of CD4^+^CD25^−^ cells resuspended at 2 × 10^6^ cells/ml in X-Vivo15 serum-free medium in the anti-CD3-precoated wells in the absence of antibiotics. Anti-CD28 antibody (2 μg/ml) and TGF-β (5 ng/ml) were immediately added, and the cells were incubated for 5 days. iTregs were isolated from the cultured cells using a CD4^+^CD25^+^ Regulatory T Cell Isolation Kit and used as a positive control.

### In-vivo study design and skin transplantation

In the skin transplantation experiments, 1.5-cm^2^ full-thickness pieces of back skin of C57BL/6 mice were transplanted to the backs of BALB/c mice, fixed in place with four to six stitches, and covered with a bandage. The bandage was removed 7 days after transplantation. The skin grafts were monitored daily; grafts rejected before day 7 were considered to involve technical errors and were excluded from the study. Grafts were considered rejected when they showed at least 90% necrosis.

The experiments included 12 groups of mice (five mice per group) and involved three kinds of treatment. Some mice received 10^6^ MSCs intravenously on days − 1 and 0 followed by days 2 and 4 after completion of the skin graft procedure. Some mice also received CsA (gavage, 50 mg/kg/day) daily beginning 7 days before transplantation to the time of sacrifice. The control group received PBS. The mice were sacrificed on day 5, 10, or 15, and CD4^+^ T cells were isolated from the splenocytes of the grafted mice for biological analysis. Sera from the animals were also used for cytokine assays.

#### Quantification of cytokines

Supernatants were collected, centrifuged, and assessed using a mouse Th1/Th2/Th17/Th22–13 Plex cytokine assay kit according to the manufacturer’s instruction (eBiosciences, USA) using a flow cytometer (FACS Calibur, BD, USA).

For in-vivo experiments, we assessed cytokine levels in the sera of skin-transplanted mice and compared them with those of the control group at days 5, 10, and 15. These days were selected based on the median survival time (MST). Flowcytomix™ Pro 3.0 software was used to calculate the sample concentrations. Standard curves (ranging from 2000 to 2743 pg/ml) for all of the analyzed cytokines were included in each run.

### Suppression assay

For the in-vitro suppression assay, we used the basic protocol described by Collison and Vignali [15] and a BrdU kit according to the manufacturer’s instructions (Cell Proliferation ELISA, BrdU). Briefly, MSCs and T cells were cultured for 72 h under different in-vitro conditions. For each condition, 10^4^ MSC-cultured T cells (C57BL/6) were added to 10^5^ CD4^+^CD25^–^ T cells (C57BL/6) (ratio 1:10) in a plate containing 10^4^ allogeneic DCs (Balb/C) and incubated for 48 h at 37 °C. Then, BrdU was added to the cultures, and the cultures were incubated for an additional 24 h. The labeling medium was then removed by centrifugation and flicking and the plates were dried at 60°C for 1 h. The cells were fixed using fixDenat and stored at 25°C for 1 h; the fixDenat was then removed by flicking and tapping. Anti-BrdU-POD was added to the plates, and the plates were incubated at 25°C for 90 min. The conjugated antibody was removed by flicking and by rinsing the plates three times with PBS. After the addition of substrate solutions and color development, the absorbance of the samples was measured in an ELISA reader at 370 nm (reference wavelength 429 nm).

For in-vivo suppression assays, CD4^+^ T cells were isolated from the spleen of skin-transplanted mice and added to effector T cells and DCs as described for the in-vitro procedure.

### RNA extraction and real-time PCR

RNA was isolated from CD4^+^DC25^+^ Tregs cultured in vitro and from CD4^+^ T cells obtained from the spleens of skin-grafted mice at the indicated times using TRIzol (Invitrogen, USA). Synthesis of cDNA synthesis was then performed using a reverse transcription kit (Takara, Japan). Quantitative RT-PCR experiments were performed using ABI Step One Plus (Applied Biosystems, USA) with GAPDH as a housekeeping gene. The specific primers for cDNA synthesis were as follows: TRAF6, forward 5′-CCTCATCAGAGAACAGATGCCTA-3′, reverse 5′-TGTCGTGCCAAGTGATTCCT-3′; STUB1, forward 5′-CATATCTCACCAGGCTCATTGC-3′, reverse 5′-TATCTGCCATGTATTTATCGTGCTTG-3′; UBC13, forward 5′-CGGAGACAAGAGCAGAGGC-3′, reverse 5′-ACGCTGGGTTTCCTTGATGA-3′; GRAIL, forward 5′-TGGGAATTGAGGTGGATGTTGAA-3′, reverse 5′-GTGGCTCATCTGCTCCTTGTA-3′; USP7, forward 5′-CTTGAATTACTGTGGACATATCTAC-3′, reverse 5′-TCGGCTTAACTTCCTCATAG-3′; FOXP3, forward 5′-AATAGTTCCTTCCCAGAGTTCTTC-3′, reverse 5′-ATGGTAGATTTCATTGAGTGTCCT-3′; and GAPDH-specific primers as internal control, forward 5′-CGGTGTGAACGGATTTGGC-3′, reverse 5′-GTGAGTGGAGTCATACTGGAAC-3′. The primers were designed using Allele ID software (version 7.5).

### Methylation analysis

CD4^+^ T-cell genomic DNA was purified with the Trizol. Methylation analysis was performed by bisulfite conversion of genomic DNA using the MethylCode™ Bisulfite Conversion Kit (lifetechnologies). The Methylation-specific and demethylation-specific amplification primers were chosen as suggested by Kim and Leonard [31]. Then, high-resolution melting (HRM) was performed using MeltDoctor™ HRM Master Mix (Thermo Fisher, USA) and Pre-mixed Calibration Standard (epigend, USA) to investigate TSDR methylation.

### Statistical analysis

In-vitro and in-vivo data were analyzed using Prism version 5 (GraphPad software). Kaplan–Meier survival curves were compared using the long-rank test. Student’s *t* test or one-way ANOVA with post-hoc comparison and two-way ANOVA analyses were performed depending on the number of comparatives. The data are represented as the mean ± SEM; *n* = 4 independent experiments. Significance levels are indicated at *p* < 0.05, *p* < 0.01, and *p* < 0.001. The significance levels of the correlation coefficients are indicated as P*** (0.8 < CC < 1), P** (0.6 < CC < 0.8), and P* (0.4 < CC < 0.6); correlation coefficients less than 0.4 were considered nonsignificant. A minus sign preceding the correlation coefficient indicates a negative correlation.

## Results

### MSCs can convert conventional T cells into Foxp3-expressing Tregs with strong immunosuppressive capacity

In the present study, using four in-vitro experimental conditions that allow Treg induction in the presence of MSCs, as described in Methods, we investigated the capacity of BM-MSCs to convert CD4^+^CD25^−^ T cells to iTregs. MSCs were obtained from the bone marrow of BALB/c mice. The MSC phenotype of the cells was confirmed by Sca-1 and CD44 membrane expression and by the absence of CD34 and CD45 markers (Additional file 1: Figure S1A) as well as by their capacity to differentiate into osteocytes and adipocytes under appropriate differentiation conditions (Additional file 1: Figure S1B). CD4^+^CD25^−^ T cells (C57BL/6) (Fig. 1a) and DCs (BALB/c) were isolated from mice spleens and cultured alone, or in cell–cell contact with MSCs (BALB/c), and under Transwell conditions for 72 h and 5 days as described in Methods. The viability of the cells under all conditions except the MSC + TC condition, in which it was 77%, was greater than 98% on day 5 (Additional file 1: Figure S2). Thereafter, the expression of the CD25^+^Foxp3^+^ population among the total CD4^+^ T cells was evaluated after 72 h and 5 days. After 72 h of culture, we observed only a modest induction of Tregs under the MSC + MLR and MSC + MLR + LPS conditions (18 ± 0.37% and 17.9 ± 0.58%, respectively) compared to the MSC + TC condition (40.5 ± 0.45%) (Fig. 1b). However, the percentage of induced Tregs in the MSC + TC group was not stable as it decreased to approximately 8.66 ± 0.15% at day 5 of coculture. By contrast, the percentage of iTregs in the MSC + MLR and MSC + MLR + LPS cultures continued to increase between 72 h and 5 days (20.12 ± 0.41% and 19.3 ± 0.96%, respectively). When the isolated DCs were cocultured with autologous MSCs for 24 h and then added to total allogeneic CD4^+^CD25^−^ T cells, we detected 45.3 ± 1.05% Tregs in the culture at 72 h and 49.3 ± 2.05% Tregs after 5 days of coculture (Fig. 1b). The Foxp3 mRNA levels in the cells were measured by RT-PCR at 6 h, 12 h, 24 h, 48 h, 72 h, and 5 days of coculture and compared with the levels of these mRNAs in iTregs obtained by classical in-vitro T-cell activation in the presence of TGF-β and IL-2 for 5 days (positive control) and with those of CD4^+^CD25^−^ T cells isolated from allogeneic MLR after 5 days of culture (negative control). We observed that coculture with MSCs induced Foxp3 mRNA expression (Fig. 1c). Foxp3 mRNA induction did not require only cell–cell contact with MSCs since we observed the same set of results but of less significance in Transwell conditions (Additional file 1: Figure S3A). Except for the MSC + TC condition on day 5, the amount of Foxp3 mRNA expression was equal to or greater than that in TGF-β iTregs at day 5 in other conditions. We observed the highest level of Foxp3 mRNA expression after 72 h in the MSC + TC condition, although Foxp3 mRNA expression in this condition decreased significantly at day 5. The level of mRNA expression in the MSC-DC condition was the highest compared with the other groups at day 5.

To investigate whether the BM-MSC-induced Tregs are as potent as TGF-β iTregs in suppressing effector T cells in the presence of allogenic DCs, total CD4^+^ T cells were harvested from cells grown under the four experimental conditions after 72 h of coculture and transferred to a new suppression assay. In all experimental conditions, the isolated CD4^+^ T cells significantly suppressed responder CD4^+^CD25^−^ T cells compared with controls when added at a 1/10 ratio; the highest suppression rate was observed for the MSC-DC condition after 48 h (Fig. 1d and Additional file 1: Figure S3B).

Due to different numbers of Tregs in each condition we performed additional experiments in which MSCs and T cells were cocultured for 3 days in all conditions. Total T cells (cells in suspension) were then collected and the CD4^+^CD25^+^ population was isolated using the Treg isolation kit. These newly induced Tregs were further put in a new MLR using freshly isolated CD4^+^CD25^−^ responder T cells in a 1/10 ratio (Treg/Tconvs) in the presence of anti-CD3 and anti-CD28 activation antibodies. We observed that Tregs induced by the MSC-DC condition represent the highest suppressive activity compared to other conditions (Fig. 1e). Therefore, these observed results are not due to a higher cell number present in their system, but due to a superior functionality of the cells produced.

### Modification of the expression of ubiquitination genes in MSC-induced Tregs

**Fig. 2:**
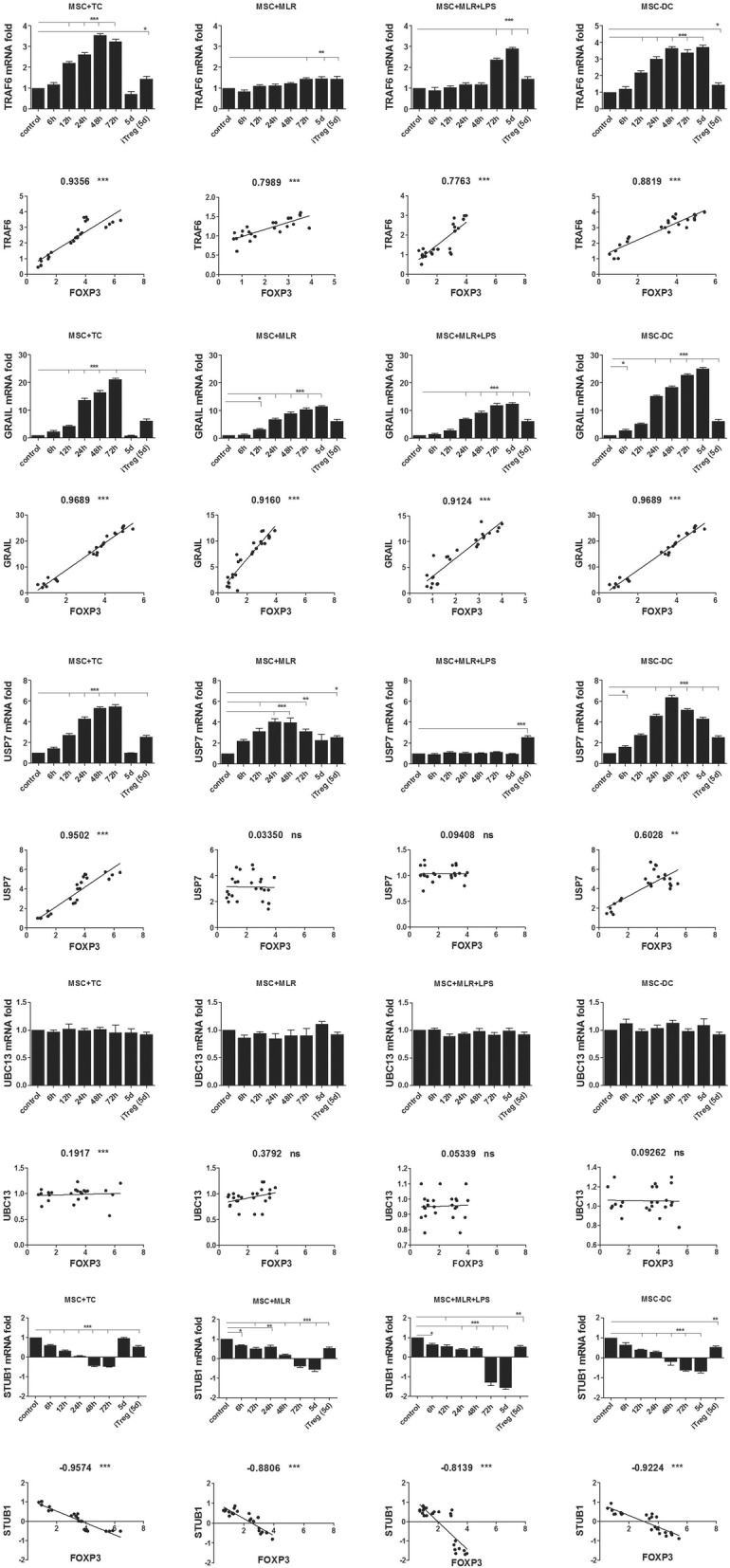
Modification of expression of ubiquitination genes in MSC-induced Tregs. CD4^+^CD25^−^ effector T cells and DCs isolated and cocultured with allogeneic MSCs under four conditions as described in Methods. Total mRNA extracted from MSC-induced Tregs after 6 h, 12 h, 24 h, 48 h, 72 h, and 5 days of coculture, and mRNA expression of TRAF6, GRAIL, USP7, UBC13, and STUB1 assessed by quantitative RT-PCR. Allogeneic MLR performed; CD4^+^CD25^−^ effector T cells isolated 5 days after MLR used as negative control, and TGF-β-induced Treg cells used as positive control. mRNA samples normalized to expression of endogenous housekeeping gene (GAPDH) and compared with negative control. Data presented as mean ± SEM; *n* = 4 independent experiments. Significant results: *p < 0.05; **p < 0.01; ***p < 0.001. Correlation of mRNA expression of each gene with that of FOXP3 mRNA shown under expression graph. Spearman correlation coefficient r and significance levels shown at top of graph. Significance levels of correlation coefficients: P*** (0.8 < CC < 1), P** (0.6 < CC < 0.8), and P* (0.4 < CC < 0.6); correlation coefficients less than 0.4 considered nonsignificant. A minus sign preceding correlation coefficient indicates negative correlation. d day, DC dendritic cell, iTreg induced Treg, LPS lipopolysaccharide, MSC mesenchymal stem cell, MSC-DC MSC-treated DC, MLR mixed lymphocyte reaction, ns not significant, TC allogeneic CD4^+^CD25^−^ T cells

Post-translational modification of the Foxp3 protein via ubiquitination could result in the preservation of Foxp3 in Tregs and maintain tolerance. We therefore investigated the mRNA expression levels of various genes that have been implicated in the ubiquitination/deubiquitination of Foxp3 under the four experimental conditions described in Methods. After 6 h, 12 h, 24 h, 48 h, 72 h, and 5 days of culture, mRNA was extracted from MSC-induced Tregs and compared with mRNA from effector CD4^+^CD25^−^ T cells that were isolated from allogeneic MLR after 5 days (negative control) and also with mRNA from iTregs generated with TGF-β and IL-2 in vitro after 5 days (positive control). The mRNA levels in the experimental samples were normalized to that of an endogenous housekeeping gene (GAPDH). As expected, TGF-β iTregs expressed higher amounts of Foxp3, TRAF6, GRAIL, and USP7 mRNAs, an equal amount of UBC13 mRNA, and lower amounts of STUB1 mRNA than the effector CD4^+^CD25^−^ T cells (Fig. 2). Interestingly, the differences were even more marked for MSC-induced Tregs compared with TGF-β-induced Tregs, and they increased with time in coculture regardless of the Treg induction method (Fig. 2). In other words, the mRNA expression of genes involved in suppressive features of Tregs, such as TRAF6, GRAIL, and USP7, is higher in MSC-induced Tregs than in iTregs generated using TGF-β and IL-2; also, the expression of STUB1 in MSC-induced Tregs is lower than that in iTregs under the other experimental conditions.

In contrast to the high levels of USP7 mRNA expression under MSC + TC, MSC + MLR, and MSC-DC conditions, we observed no increase in USP7 expression under MSC + MLR + LPS conditions. It is worth noting that decreased expression of USP7 in the presence of LPS was previously observed [58, 63]. Again, we did not observe any major changes in the expression of UBC13 mRNA in MSC-induced Treg populations under any of the conditions tested.

Importantly, according to the correlation coefficients, we always observed a strong correlation between the mRNA expression of TRAF6, GRAIL, and USP7 and the mRNA expression of Foxp3. The expression of STUB1 mRNA also showed a significant negative correlation with the level of Foxp3 mRNA (Fig. 2). We confirmed these observations by performing the same experiments using Transwell conditions (Additional file 1: Figure S4). In cells grown under Transwell conditions, we observed an increase in the mRNA expression of TRAF6 and GRAIL, no change in the level of UBC13 mRNA, and a reduced level of STUB1 mRNA under all conditions. Increased USP7 mRNA expression was observed in the MSC + TC, MSC + MLR, and MSC-DC conditions, whereas the USP7 mRNA level was unchanged in the MSC + MLR + LPS condition. The changes in the mRNA expression of the mentioned genes under Transwell culture conditions were less marked than the changes that were observed in the coculture systems (Additional file 1: Figure S4).

Overall, under most of the tested in-vitro conditions, we observed increased levels of TRAF6, GRAIL, and USP7 but not UBC13 mRNAs. TRAF6, GRAIL, and UBC13 have all been implicated in the protective ubiquitination process, whereas USP7 is thought to be involved in the protective deubiquitination process that leads to the preservation of Foxp3 expression. However, we observed decreased levels of STUB1 mRNA, which is thought to be involved in deubiquitination leading to Foxp3 protein degradation.

### BM-MSCs induce regulatory T cells with demethylated TSDR under coculture conditions

A stable phenotype of Tregs is observed when the TSDR is demethylated. As we observed enhanced expression of suppressive genes in MSC-induced Tregs and due to the important role of TSDR methylation in Treg stability, we investigated whether coculture with BM-MSCs induces TSDR demethylation. To this end, total CD4^+^ T cells cocultured with MSCs under all conditions were harvested, the genomic DNA of the cells was isolated, and the bisulfite conversion method was performed. The TSDR was amplified by RT-PCR with methylation-specific primers, and the HRM technique was performed to determine the exact melting temperature of the TSDR under each condition. The presence of more methylated CpG increases the melting temperature of the DNA.

**Fig. 3:**
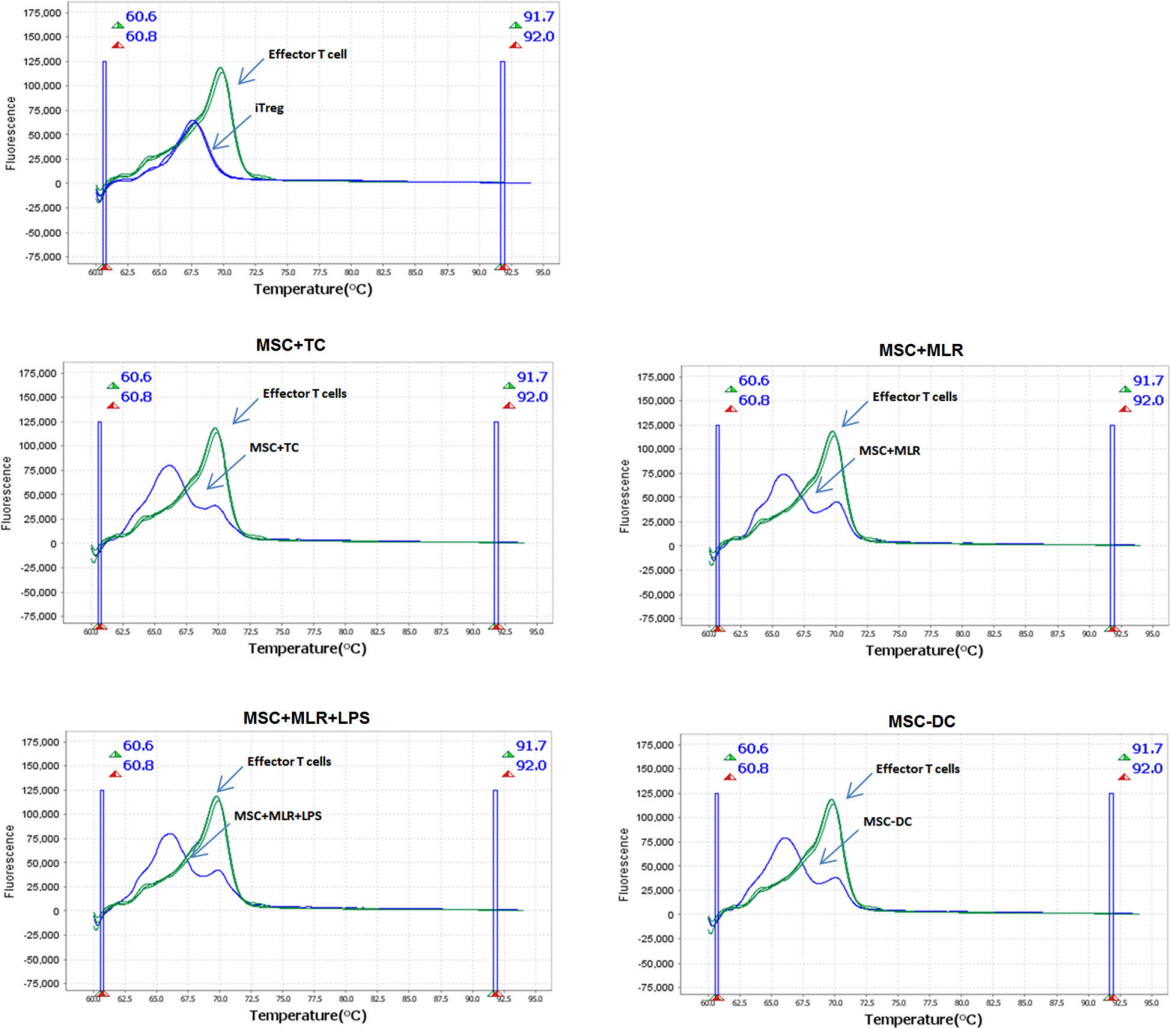
BM-MSCs enhance demethylation of TSDR in coculture system. CD4^+^ effector T cells and DCs isolated and cocultured with MSCs under four conditions as described in Methods. Genomic DNA extracted from MSC-cultured T cells after 72 h, and TSDR methylation assessed by bisulfite conversion and HRM as described in Methods. Allogeneic MLR performed; CD4^+^CD25^−^ effector T cells isolated 5 days after MLR used as negative control, and TGF-β-induced Tregs used as positive control. DC dendritic cell, iTreg induced Treg, LPS lipopolysaccharide, MSC mesenchymal stem cell, MSC-DC MSC-treated DC, MLR mixed lymphocyte reaction, TC allogeneic CD4^+^CD25^−^ T cells

We observed a single temperature peak for effector T cells in the MLR (negative control) that represents a single population in the TSDR of Foxp3. The melting temperature of this population is approximately 70 °C (Fig. 3). HRM analysis of TGF-β-induced Tregs showed a single temperature peak that represents the single TSDR population. The melting temperature of this population was approximately 67.5 °C (Fig. 3). HRM analysis of MSC-cultured T cells showed two temperature peaks; the first occurred at approximately 66.5 °C, similar to that of iTregs, and the other occurred at approximately 70 °C, like the single peak found in effector CD4^+^CD25^−^ T cells. The two peaks represent two heterogeneous populations in terms of the methylation pattern of the TSDR. We did not observe TSDR demethylation in cells cultured under Transwell conditions; in those cells, we observed a single peak at approximately 70 °C (Additional file 1: Figure S5).

### MSCs reduce the production of proinflammatory cytokines and increase IL-2 and IL-10 secretion

**Fig. 4:**
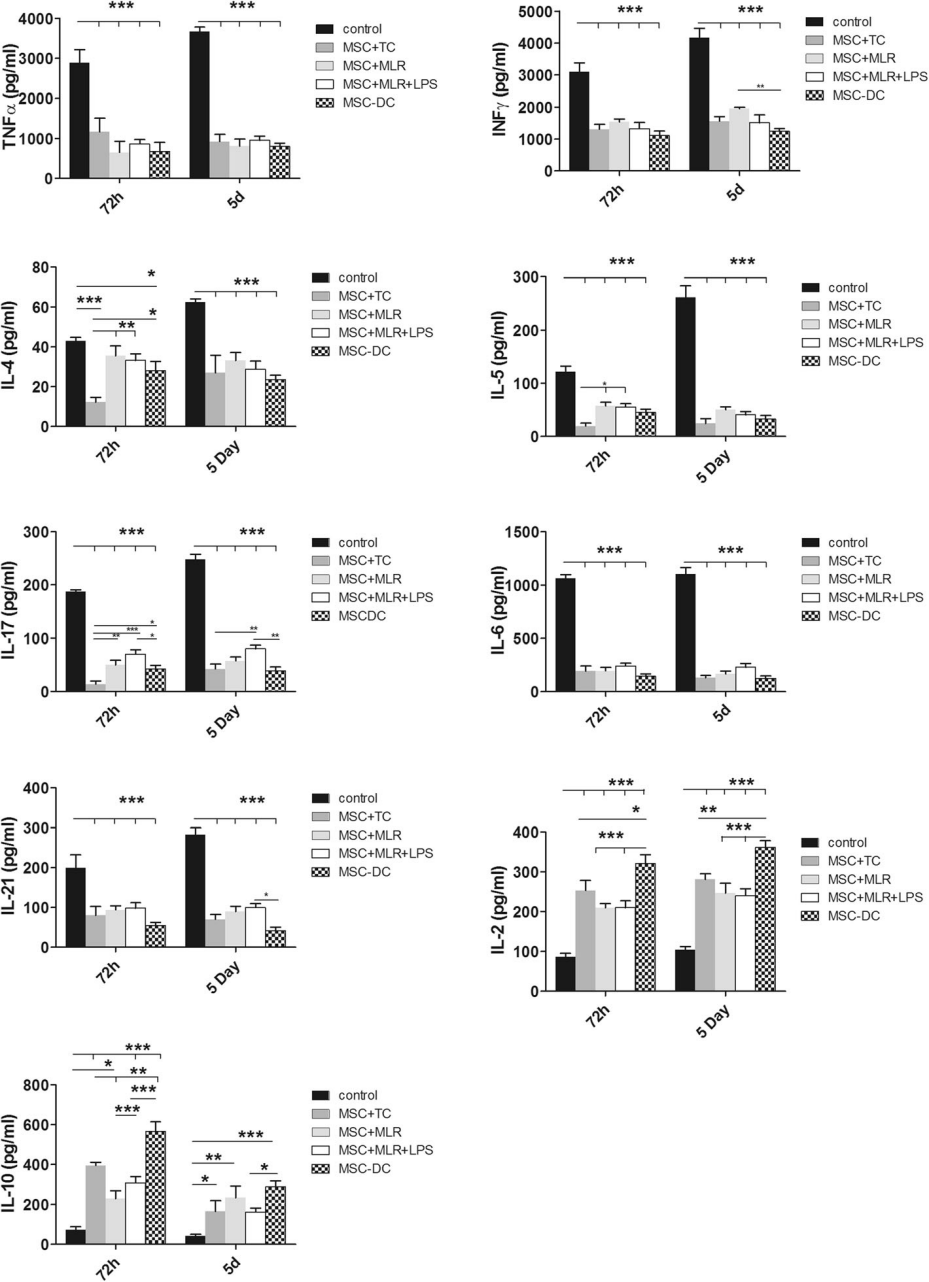
MSCs reduce proinflammatory cytokine production and increase IL-2 and IL-10 production. CD4^+^ effector T cells and DCs isolated and cocultured with MSCs under four conditions as described in Methods. Supernatant collected, and levels of TNF-α, INF-γ, IL-4, IL-5, IL-17, IL-6, IL-21, IL-2, and IL-10 measured after 72 h and 5 days of coculturing by flow cytometry and compared with negative control. Data presented as mean ± SEM; *n* = 4 independent experiments. Significant results: *p < 0.05; **p < 0.01; ***p < 0.001. d days, DC dendritic cell, IL interleukin, INF interferon, iTreg induced Treg, LPS lipopolysaccharide, MSC mesenchymal stem cell, MSC-DC MSC-treated DC, MLR mixed lymphocyte reaction, TC allogeneic CD4^+^CD25^−^ T cells. TNF tumor necrosis factor

We next aimed to evaluate the inflammatory context of the cell culture conditions by quantifying cytokine levels in cell culture supernatants obtained after 72 h and 5 days of coculture of CD4^+^CD25^−^ T cells with MSCs under the four experimental culture conditions. TNF-α and INF-γ were intensively produced by activated effector T cells in the control group (Fig. 4). We observed that compared to conventional T cells cultured with allogeneic DCs (negative control), coculture with MSCs induced a dramatic reduction in TNF-α and INF-γ secretion under all experimental conditions. It has been suggested that TRAF6 reduces both IL-4 and IL-5 production [42], whereas GRAIL reduces IL-17 and IL-21 production [45]. In addition, IL-6 represents an important checkpoint gene that controls the differentiation of cells into Tregs or Th17 T cells [9]. We observed that in the presence of MSCs the levels of all of the cytokines that are involved in T-cell differentiation were significantly reduced (Fig. 4). The secretion of inflammatory cytokines decreased within 72 h of coculture and remained at low levels at day 5. By contrast, IL-2 production was significantly increased in the presence of MSCs, in accordance with previous reports showing a strong dependency of Tregs on IL-2 [10, 13]. Finally, IL-10, an anti-inflammatory cytokine, showed a peak value after 72 h and remained significantly increased at day 5 (Fig. 4). Interestingly, cells cultured in the MSC-DC condition usually showed a more pronounced effect than cells cultured under the other conditions. Similar results were obtained with cells cultured under Transwell conditions (Additional file 1: Figure S6).

### In-vivo administration of BM-MSCs prolongs skin allograft survival

We next evaluated the capacity of BM-MSCs to suppress effector T cells in vivo and to increase graft survival in a model of fully allogeneic skin transplantation (transplantation of C57BL/6 skin into BALB/c mice). It was recently shown that low doses of MSCs in the absence of immunosuppressive agents did not prolong graft survival [3], whereas injection of high doses of MSCs prior to and subsequent to transplantation efficiently prolonged skin graft survival [64]. Therefore, we injected high doses of recipient-type ex-vivo-expanded BM-MSCs at the time of transplantation and on the 2 days following the graft (D1 and D2, MSC group). In parallel, we compared the effects of this treatment with that of cyclosporin A (CsA) treatment (CsA group) and with that of PBS treatment (PBS group). The fourth group of mice underwent autologous skin transplantation.

**Fig. 5:**
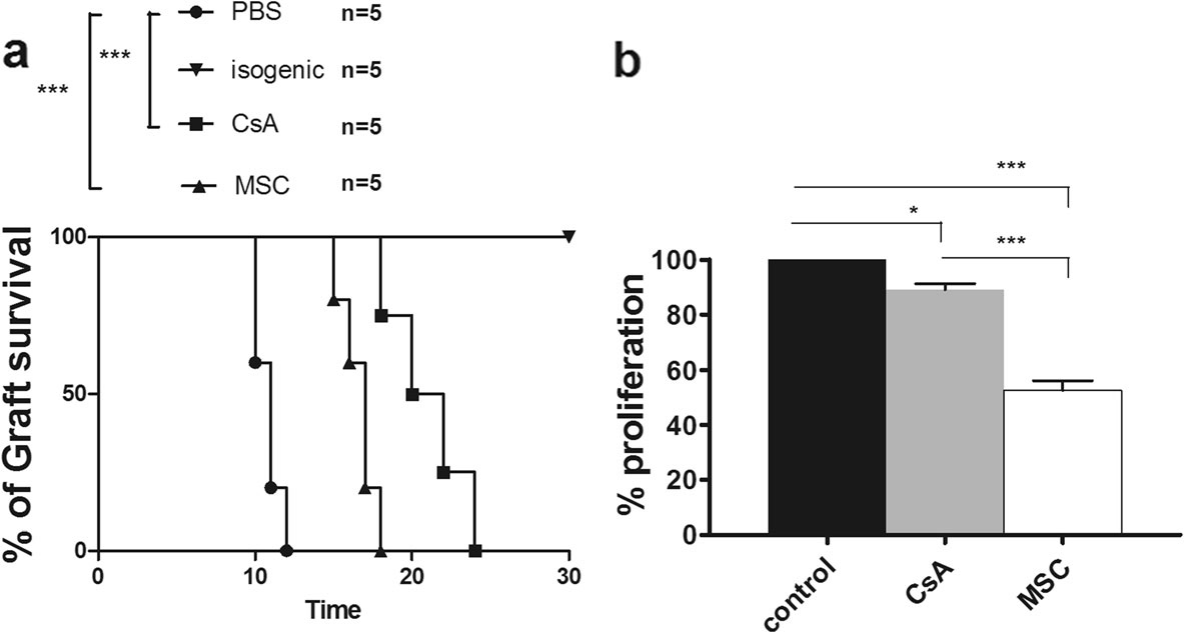
In-vivo administration of BM-MSCs prolongs skin allograft survival. BALB/c mice grafted with full-thickness allogeneic back skin from C57BL/6 mice and treated with PBS, MSCs, or cyclosporin as described in Methods. **a** Survival graph of transplanted mice showed median survival time (MST) of MSC-treated mice is 17 days (*n* = 5), of cyclosporin-treated animals is 21 days (*n* = 5), and of PBS-treated animals (*n* = 5) is approximately 11 days. **b** For in-vivo suppression assay, T cells isolated from skin-transplanted mice and added to T cells that were stimulated with allogeneic DCs. After 48 h, BrdU kit used to measure proliferation of CD4^+^ T cells, and compared with that of cells from PBS-treated mice. Data presented as mean ± SEM; *n* = 5 in each group. Significant results: *p < 0.05; ***p < 0.001. CsA cyclosporin A, MSC mesenchymal stem cell, PBS phosphate buffered saline

As expected, no graft rejection was observed in the autologous control mice, whereas the PBS-treated mice rapidly rejected their grafts (MST = 11 days) (Fig. 5a). Treatment with CsA significantly prolonged skin graft survival (MST = 21 days). In this setting, infusion of high numbers of MSCs (1 million/day for 3 days) significantly prolonged skin allograft survival compared with PBS-treated mice (MST = 17 days) (Fig. 5a). We next reproduced our allogeneic skin graft model and applied the same set of treatments. After 5 days, the recipient mice were sacrificed and their spleens were harvested. We isolated CD4^+^ T cells from their splenocytes and evaluated their suppressive ability by adding them to an autologous suppression assay. Interestingly, although CsA-treated mice showed a better protective effect than MSC-treated mice in terms of skin graft survival, T cells harvested from MSC-treated mice were remarkably more efficient in suppressing responder CD4^+^ T cells in vitro than were CD4^+^ T cells collected from PBS-treated mice (Fig. 5b).

### BM-MSC infusion delays skin graft rejection and is associated with increased Foxp3 expression and modification of ubiquitination gene expression in iTregs

**Fig. 6:**
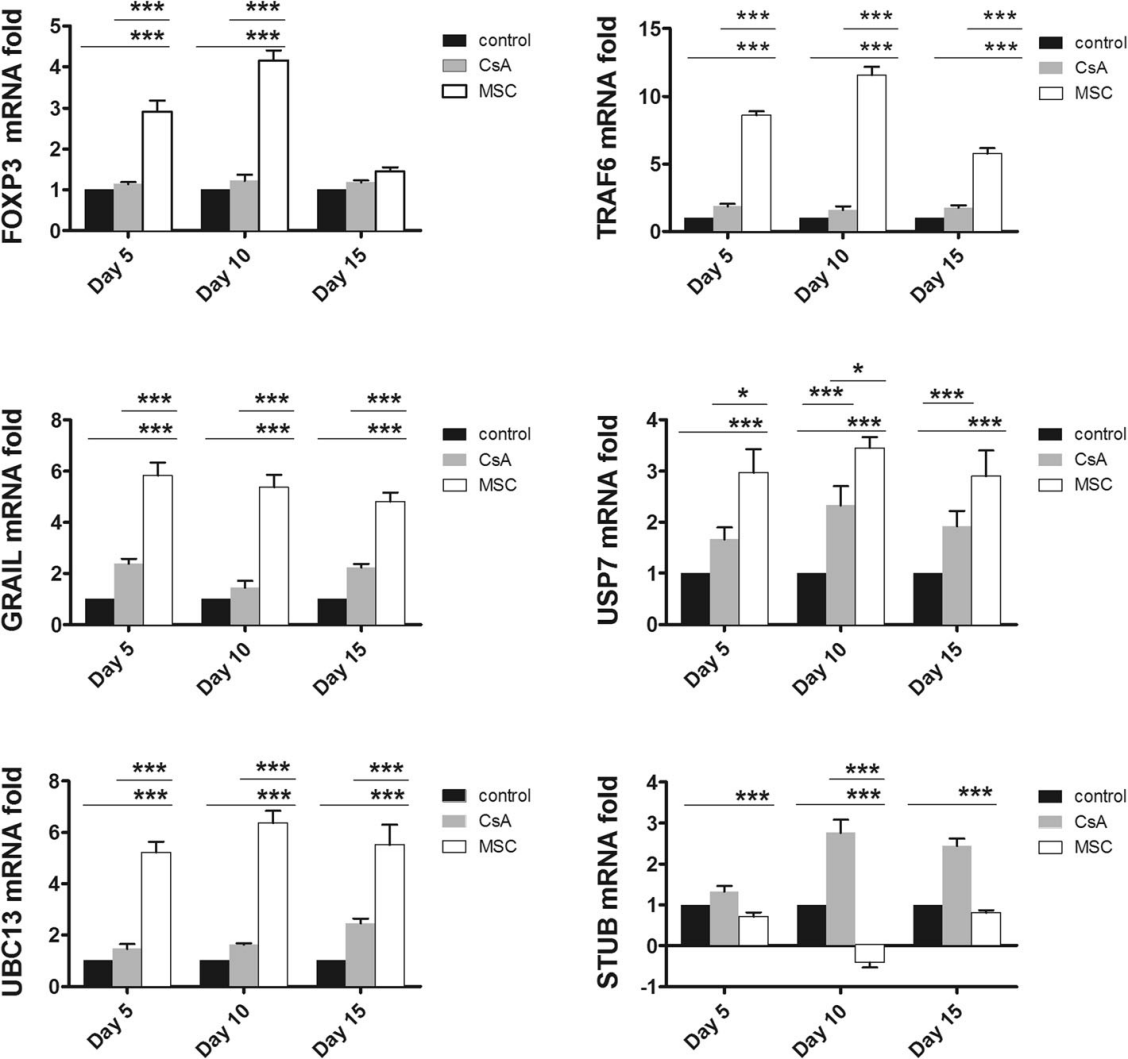
Infusion of BM-MSCs delays skin graft rejection via increased FOXP3 expression and modification of ubiquitination gene expression in iTregs. BALB/c mice grafted with full-thickness allogeneic back skin from C57BL/6 mice and treated with MSCs, cyclosporin, or PBS as described in Methods. Mice sacrificed on days 5, 10, or 15, and total mRNA extracted from splenic CD4^+^ T cells. Expression of TRAF6, GRAIL, USP7, UBC13, and STUB1 mRNA assessed by quantitative RT-PCR. mRNA samples normalized to expression of endogenous housekeeping gene (GAPDH) and compared with PBS-treated group. Data presented as mean ± SEM; *n* = 5 in each group. Significant results: *p < 0.05; ***p < 0.001. CsA cyclosporin A, MSC mesenchymal stem cell

In vitro, we were able to demonstrate that the induction of Tregs by MSCs is associated with increased Foxp3 mRNA and protein expression as well as with increased TRAF6, GRAIL, and USP7 mRNA expression and decreased STUB1 expression. Here, we investigated whether the same mechanisms were involved when MSCs were administered directly in vivo. We reproduced the allogeneic skin graft experiments, sacrificed the mice on days 5, 10, and 15, and harvested CD4^+^ T cells from their splenocytes. We extracted the total CD4^+^ T-cell mRNA from the splenocytes and measured the mRNA expression of Foxp3, TRAF6, GRAIL, USP7, UBC13, and STUB1. Interestingly, we observed that the mRNA expression of the Foxp3 gene was elevated at days 5 and 10 post grafting only in MSC-treated mice and not in the two other groups (Fig. 6). Moreover, at all three time points examined, Foxp3 mRNA expression was associated with increased mRNA expression of the TRAF6, GRAIL, USP7, and UBC13 ubiquitination genes, comparable to the increase that was observed in the in-vitro experiments (Fig. 6). The mRNA expression of TRAF6, GRAIL, USP7, and UBC13 increased only in MSC-treated mice.

It has been shown that CsA can inhibit Treg generation [61]. Interestingly, we did not observe significant changes in mRNA expression of Foxp3, TRAF6, GRAIL, USP7, or UBC13 in CsA-treated mice. In the case of STUB mRNA, not only was a remarkable decrease observed in MSC-treated mice, but a significant increase was also found in CsA-treated mice, indicating that CsA interferes with the stabilization of Foxp3 through STUB1-induced degradation of Foxp3 via proteasomes [12]. These data show that the ability of MSCs to induce Tregs in vivo may rely on the regulation of ubiquitination pathways.

### BM-MSC infusion delays skin graft rejection via modification of the cytokine secretion profile

**Fig. 7:**
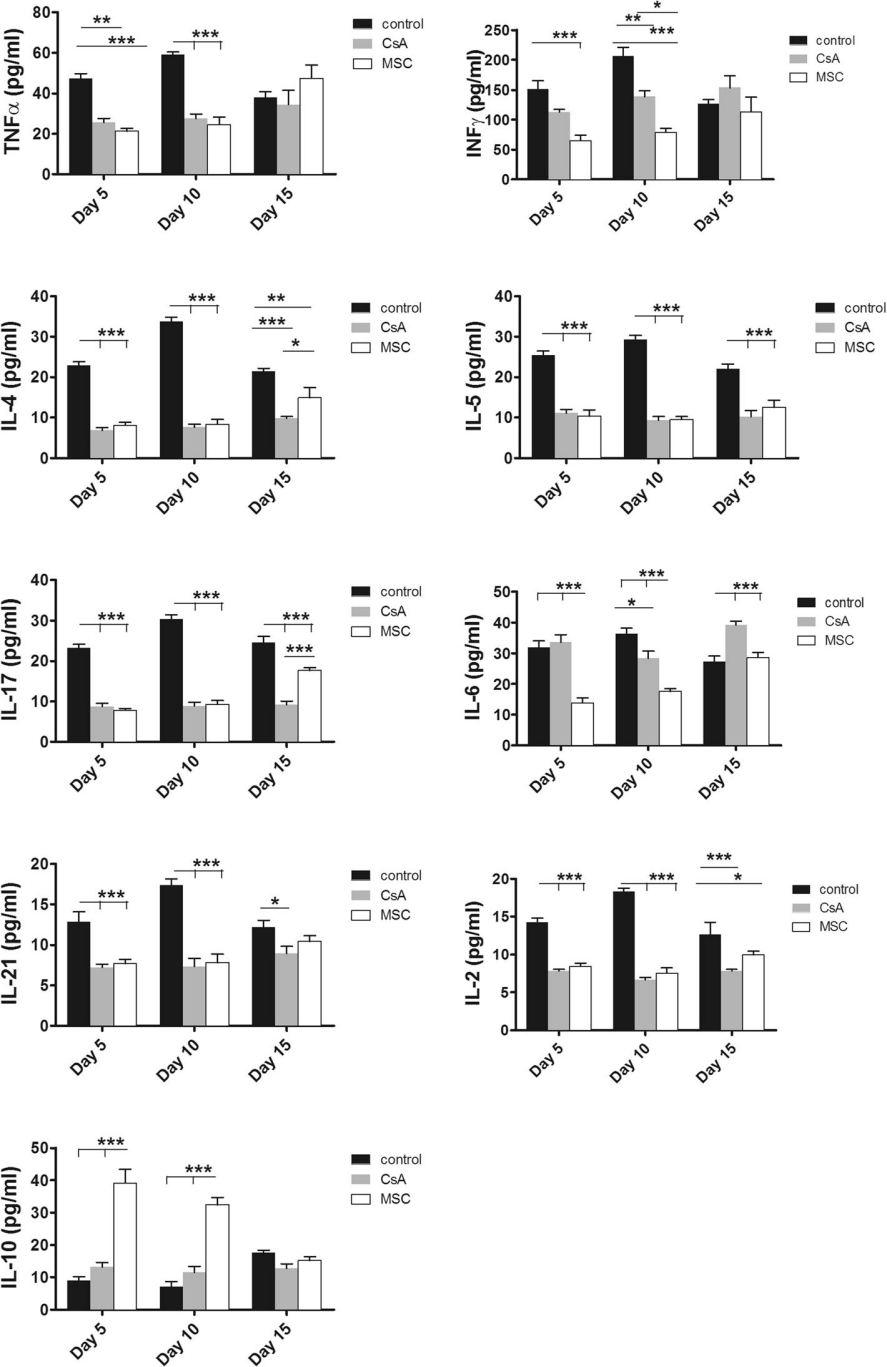
Infusion of BM-MSCs delays skin graft rejection via modification of cytokine secretion profile of T cells. BALB/c mice grafted with full-thickness allogeneic back skin from C57BL/6 mice and treated with MSCs or cyclosporin as described in Methods. Mice sacrificed on day 5, 10, or 15 after transplantation, and serum levels of TNF-α, INF-γ, IL-4, IL-5, IL-17, IL-6, IL-21, IL-2, and IL-10 measured. Data presented as mean ± SEM; *n* = 5 in each group. Significant results: *p < 0.05; **p < 0.01; ***p < 0.001. CsA cyclosporin A, IL interleukin, INF interferon, MSC mesenchymal stem cell, TNF tumor necrosis factor

Similarly, we verified whether the changes in cytokine production observed in the in-vitro experiments were also observed when skin graft rejection was delayed by administration of MSCs. At days 5, 10, and 15 following skin grafting, the levels of various cytokines were measured in serum obtained from blood samples. We observed that in both CsA-treated and MSC-treated mice, the serum levels of inflammatory cytokines such as IL-4, IL-5, IL-17, IL-21, TNF-α, INF-γ, and IL-2 were significantly reduced compared with those in PBS-treated mice (Fig. 7). Interestingly, in parallel with Foxp3 overexpression, an elevated production of IL-10 was observed in mice receiving MSCs until day 10 post grafting, whereas no modifications in IL-10 production were observed in CsA or PBS-treated mice (Fig. 7).

Together, these data demonstrate that MSCs reduce effector T-cell responses by inhibiting the production of inflammatory cytokines and enhancing the secretion of anti-inflammatory IL-10.

## Discussion

The multilineage differentiation and immunomodulation capabilities of MSCs make these somatic progenitor cells an interesting tool for cell therapy. The immunosuppressive effect of MSCs has been investigated in various animal models of autoimmune diseases, including models of experimental autoimmune encephalomyelitis (EAE) [5] and collagen-induced arthritis (CIA) [47]. In addition, MSCs are being used in tissue and organ transplantation in several clinical trials, including GVHD [4], islet transplantation [21], liver transplantation [55], and renal transplantation [8], with promising results. Approximately 500 MSC-related clinical trials were registered on the NIH Clinical Trial Database as of 2016 (https://clinicaltrials.gov/), and nearly half of these trials are based on the immunomodulatory effects of MSCs.

The ability of MSCs to convert conventional T cells to iTregs was previously observed [19, 58]. It was also previously shown that iTreg stability and suppressive functions depend strongly on Foxp3 stability. In parallel, ubiquitination/deubiquitination mechanisms were demonstrated to be implicated in Foxp3 stability and in the suppressive phenotype of Tregs [12, 58]. Here, we show for the first time that MSC-iTreg generation is associated with changes in the mRNA expression of ubiquitination genes (TRAF6, GRAIL, USP7, UBC13, and STUB1) that are involved in the suppressive phenotype of Tregs obtained from CD4^+^CD25^−^ conventional T cells. In addition, we showed that MSCs can enhance the demethylation of the TSDR only via cell–cell contact and not under Transwell conditions.

In this work, we used four different in-vitro models that promote MSCs to generate iTregs. We observed that variable amounts of iTregs were produced depending on the experimental model used. When we examined the expression of five ubiquitination genes (TRAF6, GRAIL, USP7, UBC13, STUB1) that have been implicated in the suppressive phenotype of Tregs and the preservation of Foxp3 expression, we observed that the transcription of all of these genes was globally modified in a way that leads to preservation of Foxp3 in Tregs and consequently produces a suppressive phenotype of these cells. Indeed, the addition of MSCs to CD4^+^CD25^−^ T-cell cultures markedly increases the mRNA expression of TRAF6, GRAIL, and USP7, all of which have been implicated in the direct or indirect protective ubiquitination process of Foxp3, whereas STUB1 expression decreased, thus preventing direct degradation of Foxp3. The convergence of these data, which were obtained under four different experimental conditions, highlights the robustness of our observations. Furthermore, compared to conditions involving direct contact of MSCs with CD4^+^CD25^−^ T cells, the addition of DCs that were previously cocultured with MSCs induced more Foxp3 mRNA and protein expression as well as mRNA of ubiquitination genes on day 5, which is associated with greater induction of immunosuppressive capacity. This result suggests that MSCs are able to modify DCs, which then become capable of inducing Tregs from CD4 T cells. Here again, it was under the MSC + TC and MSC-DC experimental conditions that we observed the greatest changes in TRAF6, GRAIL, and USP7 mRNA expression, suggesting that the higher the level at which these genes are expressed, the more suppressive iTregs become.

By contrast, we also observed less stable Foxp3 mRNA and protein expression under MSC + TC conditions on day 5. This effect might be due to the absence of TCR-induced T-cell signaling in the absence of DCs. The expression of FOXP3 in these experiments decreases on day 5 only in the TC + MSC condition. Indeed, T cells shift to resting status in the absence of TCR stimulation since in this condition DCs are absent. MSCs are not immunogenic since they fail to elicit a proliferative response in allogeneic lymphocytes [44, 48, 56]. Also, in-vivo investigations proved the persistence of MSCs while infused to the immunocompetent recipient after transplantation [4, 27]. MSCs do not express FAS ligand or costimulatory molecules, such as B7-1, B7-2, CD40, or CD40L. When costimulation is inadequate, T-cell proliferation can be induced by the addition of exogenous costimulation. Even in presence of CD28-stimulating antibody and HLA-mismatched lymphocytes, no T-cell proliferation can be observed [56]. Hence, according to these data and also Additional file 1: Figure S2, it seems that T cells could not survive in the absence of stimulation. It can be argued that the CD4^+^ population isolated from the MSC-DC group at day 5 contains twice the number of FoxP3-expressing cells as the CD4^+^ populations isolated from the MSC + MLR and MSC + MLR + LPS groups. Thus, the possibility that the increased suppression that was observed is a cell number effect caused by the higher abundance of cells with Treg phenotypes and is not due to the enhanced functionality of these cells cannot be excluded. However, the increased suppression observed under the MSC + TC and MSC-DC conditions was invariably correlated with changes in mRNA expression that are compatible with increased Foxp3 stabilization. MSCs are not immunogenic, and they fail to elicit a proliferative response in allogeneic lymphocytes [48]. In addition, in-vivo investigations have shown that MSCs persist when infused into immunocompetent recipients after transplantation [4]. MSCs do not express the FAS ligand or costimulatory molecules such as B7-1, B7-2, CD40, or CD40L [56]. When costimulation is inadequate, T-cell proliferation can be induced by the addition of exogenous costimulatory molecules. Even in the presence of CD28-stimulating antibody and HLA-mismatched lymphocytes, no T-cell proliferation is observed [56].

Importantly, the suppressive phenotype of Tregs driven by ubiquitination genes correlated with decreased production of TNF-α, INF-γ, IL-4, IL-5, IL-6, IL-17, and IL-21 proinflammatory cytokines, whereas the immunosuppressive IL-10 cytokine intensively increased as soon as 72 h. Unfortunately, the cellular source of cytokine production cannot be determined in our experimental conditions. Consequently, this point remains to be addressed using, for instance, MSCs or T cells collected from cytokine-knockout mice. However, investigation previously showed that TRAF6-deficient T cells confer an inflammatory phenotype that is leading to abnormal Th2 activation and secretion of IL-10. The increase in TRAF6 gene expression in our study is consistent with the important anti-inflammatory effects observed in our experiments.

The interpretation of increased IL-2 production could be more complex as this cytokine is classically implicated in conventional T-cell expansion/activation. Indeed, it is well known that Tregs depend strongly on IL-2 for their survival, consistent with the important immunosuppressive effects observed in our experiments.

Importantly, together with the enhanced mRNA expression of ubiquitination genes involved in the suppressive phenotype of MSC-induced Tregs, enhanced demethylation of the TSDR results in more and stable Foxp3 expression that consequently leads to more suppressive and more stable MSC-induced Tregs. The increase in methylated CpG levels leads to increased melting temperatures in the methylation assay [43]. In the coculture systems used here, we observed two TSDR populations that differ in melting temperature: a Treg population with more demethylated TSDR, and a population of effector T cells and/or Tregs in which the TSDR contains a higher proportion of methylated CpGs and hence displays a higher melting temperature. Moreover, in cells cultured in the Transwell system, we observed a single homologous TSDR population with a melting temperature similar to that of the TDSR of effector T cells in the control group. These data show that MSC-induced Tregs produced in the Transwell system are more unstable than MSC-induced Tregs in the coculture system due to the presence of a more highly methylated TSDR. This result reveals the importance of cell–cell contact in regulation of the immunomodulatory properties of MSCs and is consistent with previous data demonstrating that cell–cell contact enhances the suppressive effect of MSCs on T cells [50, 54]. In 2013, Engela et al. [20] reported that coculture with human adipose tissue-derived MSCs had no effect on TSDR methylation. The different results obtained in our study may be due to the source of MSCs, the species used, the number of cells in the cocultures, and/or the duration of culturing. Briefly, here our data showed that MSCs can induce Foxp3 expression either by cell–cell contact or cytokines, but cell–cell contact led to demethylation of the TSDR that resulted in a more suppressive and stable phenotype of Tregs, while secretion of MSCs can induce Foxp3 expression in CD4^+^ T cells but this expression does not occur along with TSDR demethylation, that results in less stable and less suppressive Tregs compared to induced Tregs in cell–cell contact.

To evaluate the importance of our results, it was essential to assess whether the mechanisms we observed in vitro also occurred in vivo. To this end, we used an immunogenic experimental model that consists of grafting allogeneic skin in immune-competent mice. Of note, it was previously observed in another experimental setting that CsA regimens resembling those used in humans are partially or not at all effective in controlling alloreactivity in vivo in rodents [62]. This finding was confirmed in our study by the observation that the MST was delayed from day 11 to day 21 in CsA-treated mice. Although less effective than CsA treatment, administration of MSCs significantly delayed graft rejection compared with untreated mice. This effect was accompanied by increased in-vitro immunosuppressive properties of the CD4^+^ T cells collected from the spleens of mice treated with MSCs compared with those of CsA-treated mice. These results are also consistent with the increase in mRNA Foxp3 expression and the changes in ubiquitination gene mRNA levels observed in mice treated with MSCs. The importance of these results is emphasized by the observations made in CsA-treated mice. As expected, Foxp3 mRNA expression did not increase in the CsA-treated group [57, 61]. Interestingly, the unchanged level of Foxp3 in this group was consistent with the insignificant changes in the mRNA levels of TRAF6, GRAIL, USP7, and UBC13, whereas STUB1 mRNA expression showed an increase in CsA-treated mice that may be associated with the reduction in Foxp3 levels by CsA.

Analysis of cytokine production also revealed a significant decrease in inflammation in mice treated with MSCs as well as an increase in the production of immunosuppressive IL-10 up to day 10. The reduction in IL-10 observed at day 15 is likely to reflect the escape of the allogeneic response from the immunosuppressive effect of MSCs. The decrease in IL-2 observed in the CsA and MSC-treated groups is more difficult to interpret. It probably reflects an inhibition of inflammation, but it is not possible in this context to evaluate the effects of such a decrease on Treg stability and function.

## Conclusions

The immunosuppressive capacities of MSCs make these cells a therapeutic tool of great potential importance in the control of immune pathologies. In the context of alloreactivity, we have shown for the first time that if MSCs have the capacity to induce Tregs from conventional T cells, they do so through interactions that induce suppressive and stable Tregs. We showed that TRAF6, GRAIL, USP7, UBC13, and STUB1 mRNA levels are altered in a manner that maintains this suppressive phenotype and that TSDR demethylation is also associated with the induction of stable Tregs. As the in-vitro and in-vivo data are similar, we assume that they reflect a real association that remains to be demonstrated in autoimmunity. Although we observed a significant increase in the expression of the mentioned genes in the presence of MSCs, additional studies in which the expression of these genes is inhibited and protein expression is evaluated are needed to confirm these mechanisms.

## Additional file

Additional file 1: **Figure S1.** Isolation and in-vitro differentiation of BM-MSCs. **Figure S2.** Viability of CD4^+^ T cells in conditions. **Figure S3.** MSCs convert conventional T cells to Foxp3-expressing Tregs in Transwell system. **Figure S4.** Modification of ubiquitination gene expression in MSCs induced Tregs in Transwell system. **Figure S5.** BM-MSCs induce regulatory T cells with methylated TSDR in Transwell system. **Figure S6.** MSCs reduce proinflammatory cytokine production but increase IL-2 and IL-10 in Transwell system (DOCX 2447 kb)

## Abbreviations

APC: Antigen-presenting cell
BM-MSC: Bone marrow-derived mesenchymal stem cell
DC: Dendritic cell
GRAIL: Gene related to anergy in lymphocytes
GVHD: Graft-versus-host disease
ILT3: Immunoglobulin-like transcript 3
iTreg: Induced regulatory T cell
MLR: Mixed lymphocyte reaction
MSC: Mesenchymal stem cell
NK: Natural killer cell
nTreg: Natural regulatory T cell
PDL-1: Programmed death-1 ligand 1
RA: Retinoic acid
STUB1: STIP1 homology and U-box-containing protein 1
Tconv: Conventional T cell
tDC: Tolerogenic dendritic cell
TNF-α: Tumor necrosis factor alpha
TRAF6: Tumor necrosis factor receptor-associated factor 6
TSDR: Treg-specific demethylated region
Ub: Ubiquitin
UBC13: Ubiquitin-conjugating enzyme 13
USP7: Ubiquitin-specific protease 7
