# Supplementary material for: Induction of CD4+CD25+FOXP3+ regulatory T cells by mesenchymal stem cells is associated with modulation of ubiquitination factors and TSDR demethylation
Source: Stem Cell Res Ther. 2018 Oct 25;9:273. doi: 10.1186/s13287-018-0991-1 (PMC6203284; doi:10.1186/s13287-018-0991-1)
Supplement: Supplementary file 1 — Figure S1. Isolation and in-vitro differentiation of BM-MSCs. Figure S2. Viability of CD4+ T cells in conditions. Figure S3. MSCs convert conventional T cells to Foxp3-expressing Tregs in Transwell system. Figure S4. Modification of ubiquitination gene expression in MSCs induced Tregs in Transwell system. Figure S5. BM-MSCs induce regulatory T cells with methylated TSDR in Transwell system. Figure S6. MSCs reduce proinflammatory cytokine production but increase IL-2 and IL-10 in Transwell system (DOCX 2447 kb) [file 13287_2018_991_MOESM1_ESM.docx]

**Induction of CD4^+^CD25^+^Foxp3^+^ regulatory T cells by mesenchymal stem cells is associated with modulation of ubiquitination factors and TSDR demethylation**

Maryam Khosravi^1,2,4^, Ali Bidmeshkipour^2^, José L. Cohen^3,4,5^, Ali Moravej ^6^, Suzzan Hojjat-Assari^7^, Sina Naserian^3,4,8,9*^ and Mohammad Hossein Karimi^1c *^

^1^Transplant Research Center, Shiraz University of Medical Sciences, Shiraz, Iran

^2^Department of Biology, Faculty of Science, Razi University, Kermanshah, Iran

^3^Université Paris-Est, UMR_S955, UPEC, F-94000, Créteil, France

^4^Inserm, U955, Equipe 21, F-94000, Créteil, France

^5^UPEC, APHP, Inserm, CIC Biothérapie, Hôpital Henri Mondor 94010 Créteil, France

^6^Noncommunicable Diseases Research Centre, Fasa University of Medical Sciences, Fasa, Iran

^7^Institut Français de Recherche et d'Enseignement Supérieur à l'International (IFRES-INT), Paris, France

^8^Inserm, U1197, Hôpital Paul Brousse, 94807 Villejuif, France;

^9^SivanCell, Alborz University of Medical Sciences, Alborz, Iran

* Sina Naserian and Mohammad Hossein Karimi are co-last authors.

^C^ Corresponding author is MHK

Tel: +98-711-6474331

Fax: +98-711-6474331

Cell Phone: +98 917 3149022

Email: Maryam3891@gmail.com

**
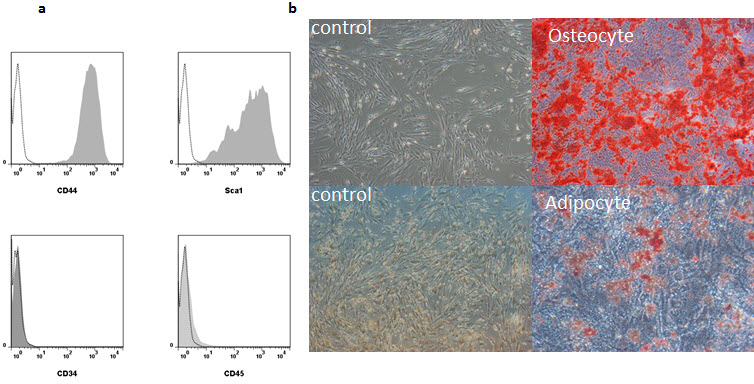
**

**FIGURE S1. Isolation and in vitro differentiation of BM-MSC.** BM-MSCs were collected and cultured by flushing from femoral and tibia bone marrow of six to eight-week old Balb/C female mice. Adherent cells were used in experiments at passage 2. (**A**) Mice BM-MSCs express surface markers such as CD44 and Sca-1and does not express specific hematopoietic markers such as CD34 and CD45. (**B**) BM-MSC differentiated into adipocyte and osteocyte cells in appropriate medium. The scale bar is 200µµm.


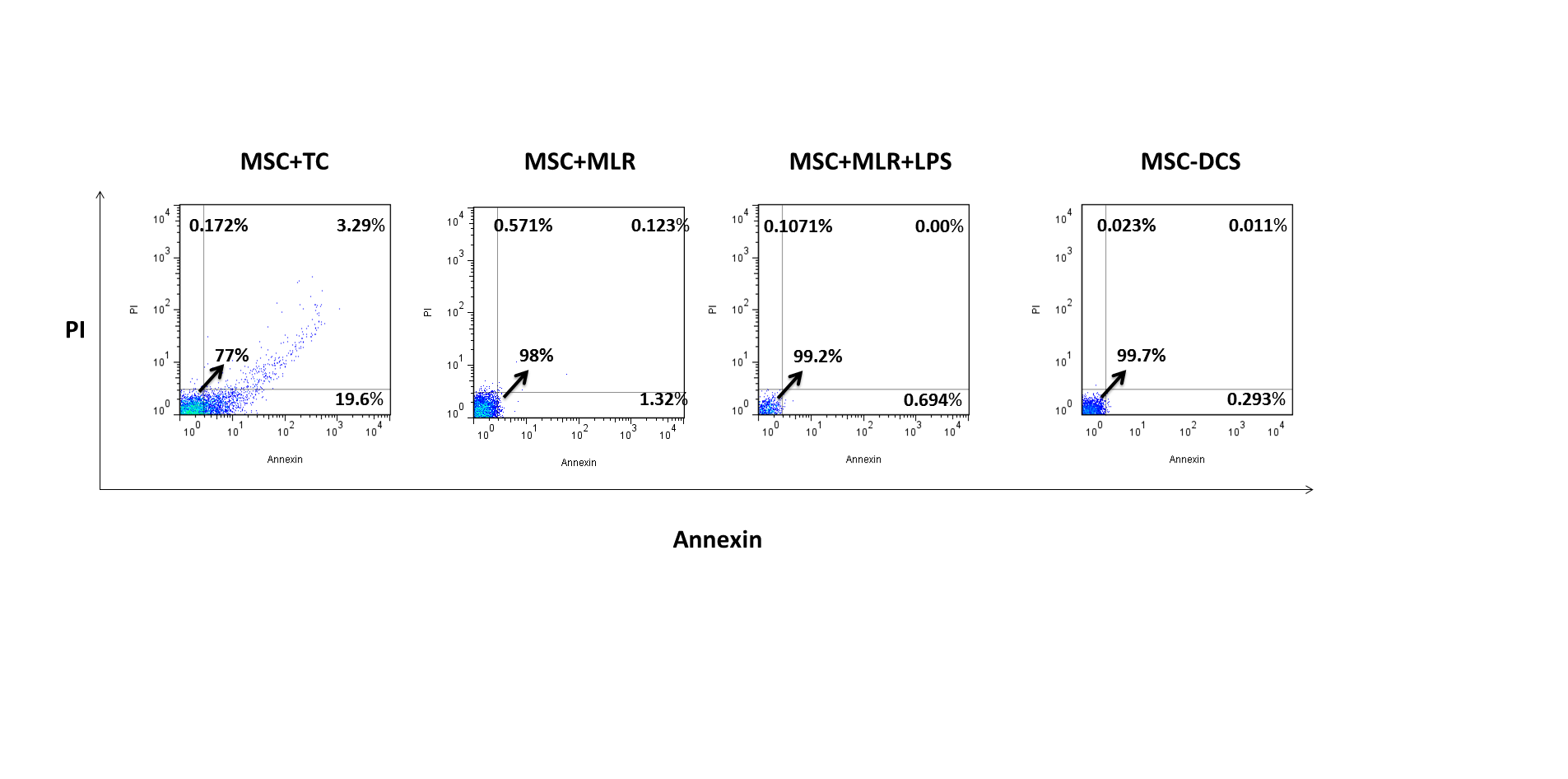


**FIGURE S2. Viability of CD4^+^ T cells in conditions.** The viability of CD4^+^ T cells were measured after 5 days of culturing with MSCs or MSC-cultured DC by annexin-PI and flowcytometry.

**
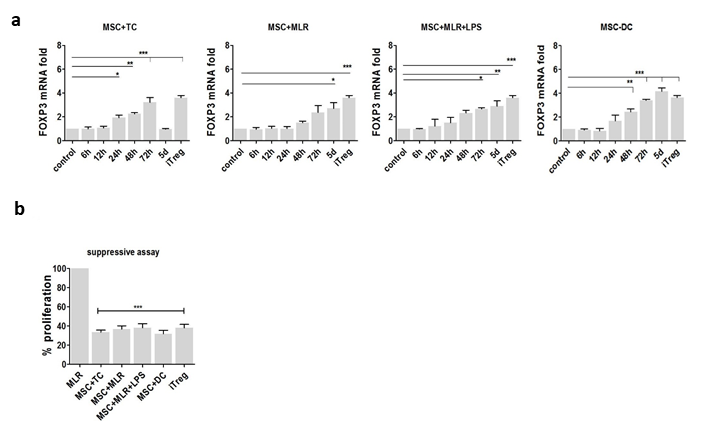
**

**FIGURE S3. MSCs can convert conventional T cells to Foxp3-expressing Tregs in transwell system.** CD4^+^ effector T cells and DCs were isolated and cultured with allogeneic MSCs in transwell system in four conditions, as described in the method section. (**A**) CD4^+^ T cells were harvested and FOXP3^+^ expression was measured by real time PCR. Allogeneic MLR was performed and CD4^+^ CD25^-^ effector T cells isolated after 5 d after MLR and used as a negative control and TGFβ- induced Treg cells were used as a positive control.The samples were normalized by expression of GAPDH and compared with the negative control. (**B**) For suppressive assay, MSC-cultured T cells were isolated after 48 h and added to T cells that were stimulated with allogeneic DCs After 48 h, BrdU was used to measure the proliferation of CD4^+^ T cells; the proliferation of those cells was compared with that of the MLR control group consisting of T cells cultured in the presence of allogeneic DCs. Data are represented as mean ± SEM; n=2 (for a) and n=4 independent experiments (b-c) and significant results as *P < .05; **P < .01; ***P < .001.

**
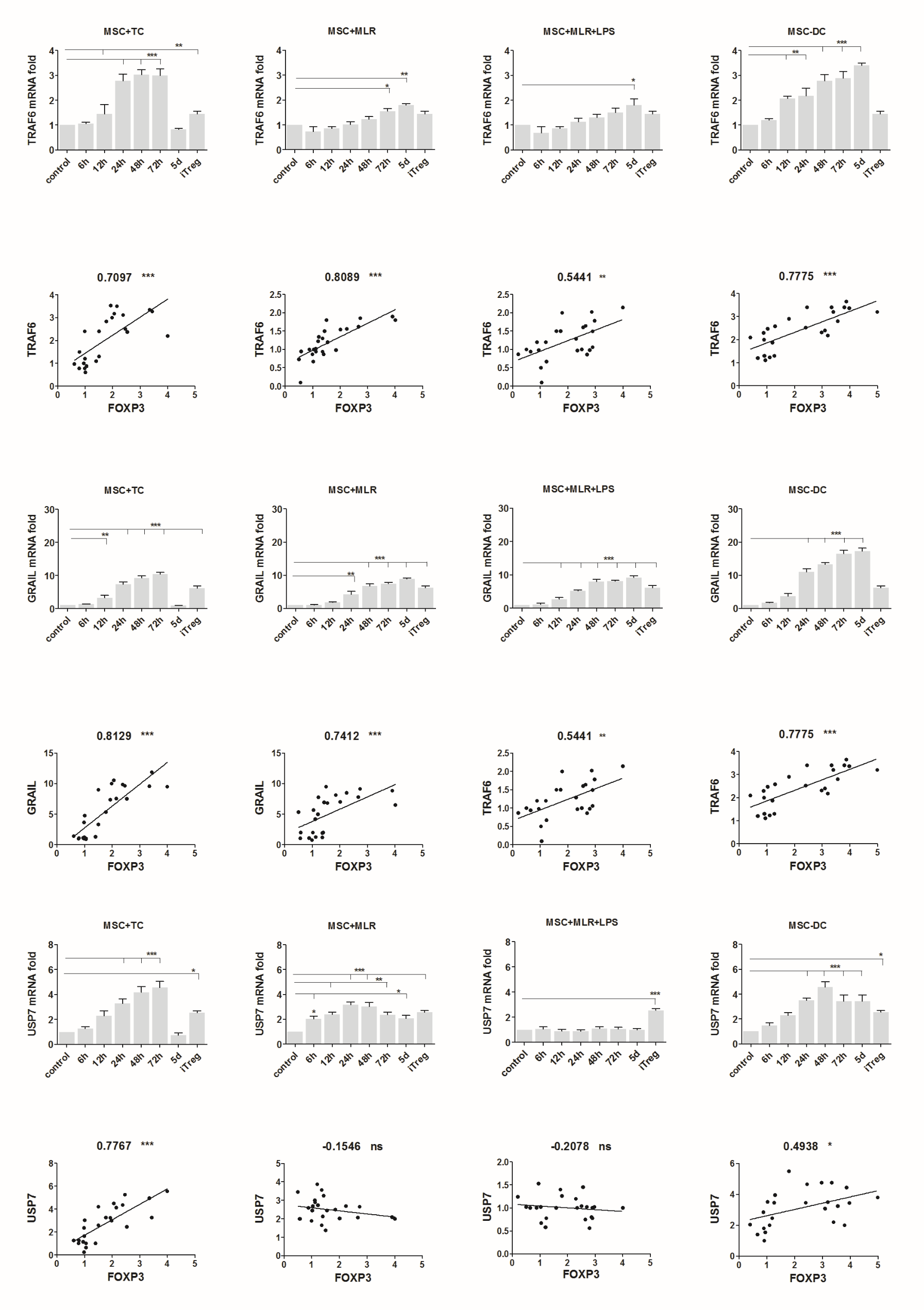
**

**
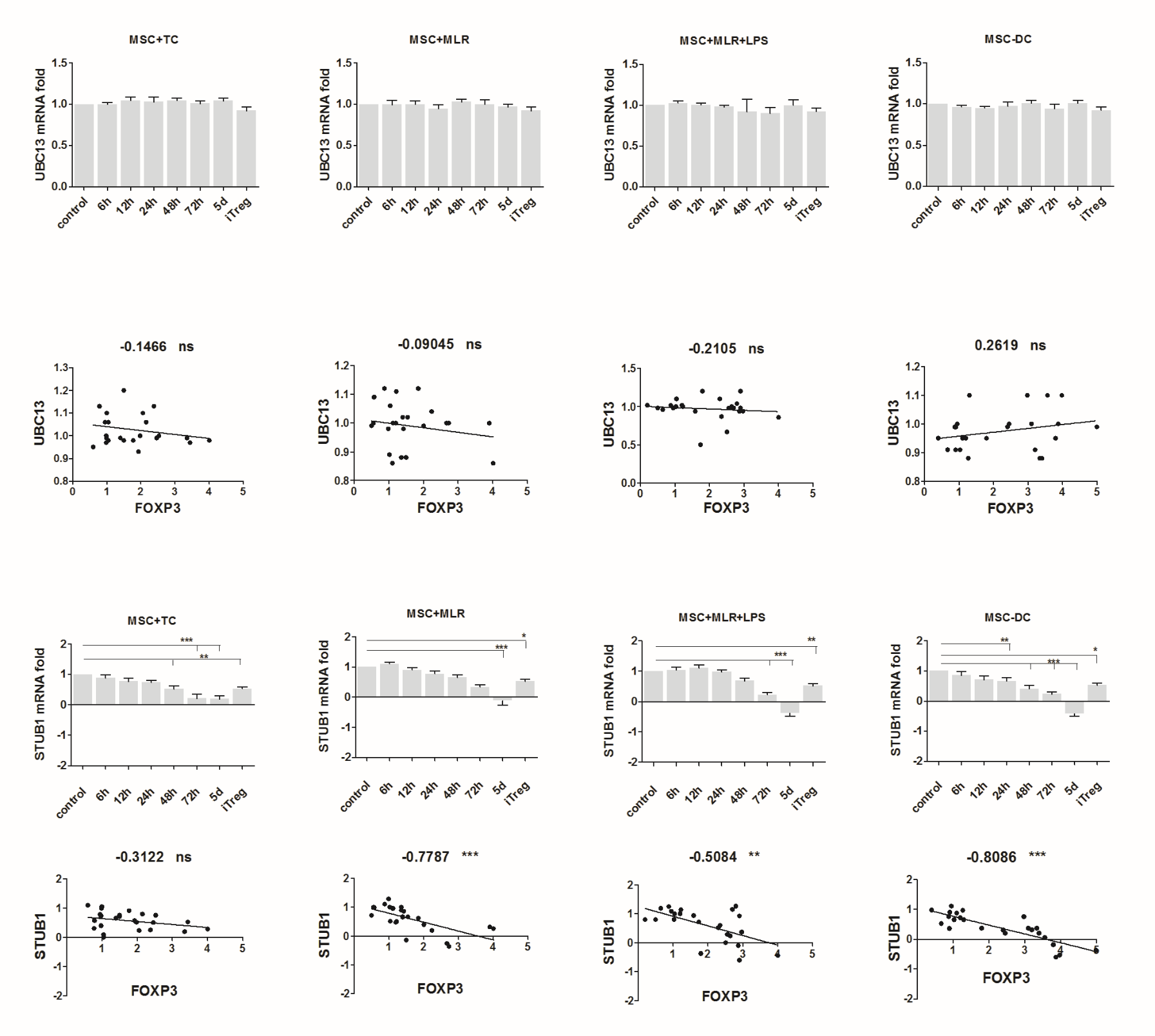
**

**FIGURE S4. Modification of ubiquitination genes expression in MSCs induced Tregs in transwell system.** CD4^+^ effector T cells and DCs were isolated and cultured with allogeneic MSCs in transwell system in four conditions, as described in the method section. Total mRNA of MSC-cultured T cells was extracted after 6 h, 24 h, 24 h, 48 h, 72 h and 5 d and expression of TRAF6, GRAIL, USP7, UBC13 and STUB1 were assessed by quantitative RT-PCR. Allogeneic MLR was performed and CD4+ CD25- effector T cells isolated after 5 d after MLR and used as a negative control and TGFβ- induced Treg cells were used as a positive control. The samples were normalized by expression of an endogenous housekeeping gene (GAPDH) and compared with the negative control. Data are represented as mean ± SEM; n=4 independent experiments and significant result as *P < .05; **P < .01; ***P < .001. Correlation of each gene with FOXP3 was shown under its expression graph. Spearman correlation coefficient r and significance levels were shown on the top of each graph. The significance level for correlations is represented as 0.8<CC<1, P***, 0.8<CC<0.6 P**, 0.6<CC<0.4 P* and CC>0.4 is considered non-significant. A minus sign preceding the correlation coefficient indicates a negative correlation**.**

**
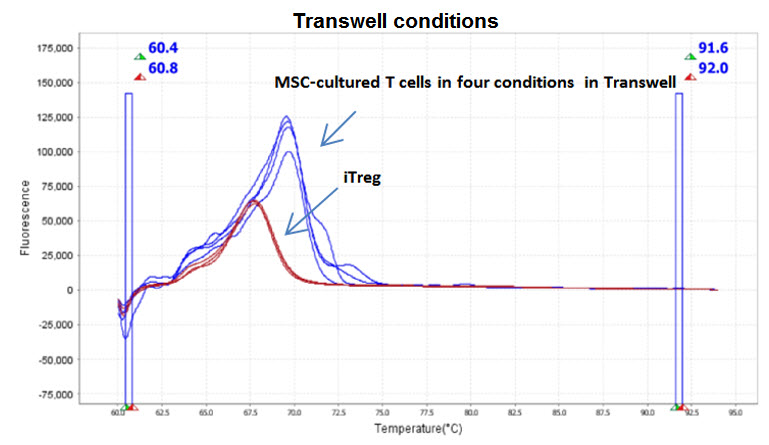
**

**FIGURE S5.** **BM-MSCs induce regulatory T cells with methylated TSDR in Transwell system.**

CD4^+^ effector T cells and DCs were isolated and cultured with allogeneic MSCs in transwell system in four conditions, as described in the method section. Genomic DNA of MSC-cultured T cells was extracted after 72 h and TSDR methylation was assessed by bisulphite conversion and HRM as describe. Normal MLR was performed as a negative control and TGFβ-induced Treg cells were used as a positive control.

**
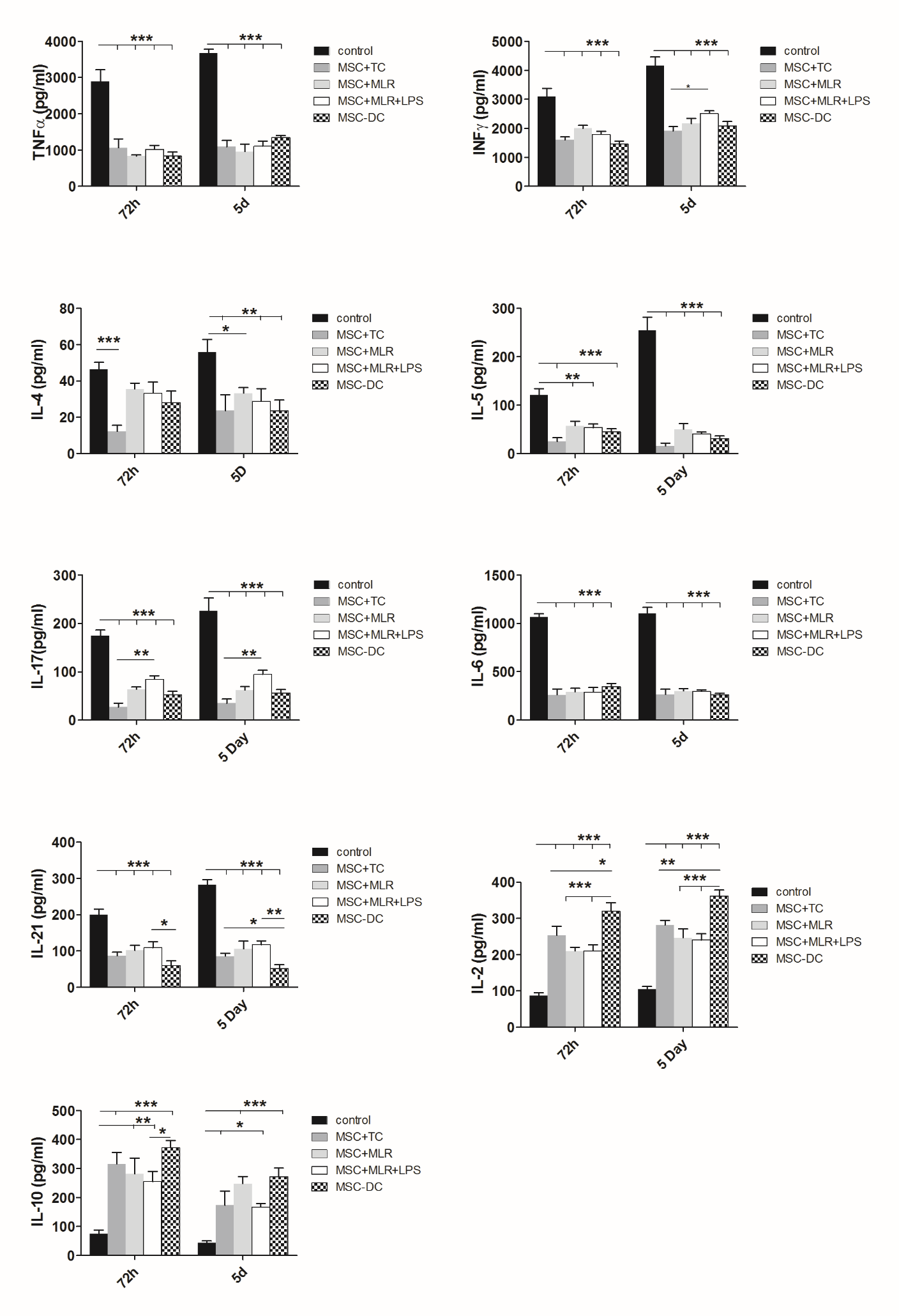
FIGURE S6. MSCs reduced pro-inflammatory cytokines production while increased IL-2 and IL-10 in transwell system.** CD4^+^ effector T cells and DCs were isolated and cultured with allogeneic MSCs in transwell system in four conditions, as described in the method section. The supernatant was collected and levels of TNFα, INF-γ, IL-4, IL-5, IL-17, IL-6, IL-21, IL-2, and IL-10 were measured after 72 h and 5 day of co-culturing, by flow cytometry and compared with the negative control. The data are represented as mean ± SEM; n=4 independent experiments and significant results as *P < .05; **P < .01; ***P < .001.
